# Mind the brain age: How segmentation and template selection reshape structural connectomes

**DOI:** 10.1162/IMAG.a.1384

**Published:** 2026-09-30

**Authors:** Carlo Ferritto, Giulia Lioi, Pierre-Yves Jonin, Julie Coloigner

**Affiliations:** Univ Rennes, CNRS, Inria, Inserm, IRISA UMR 6074, EMPENN — ERL U 1228, Rennes, France; IMT Atlantique, Lab-STICC UMR CNRS 6285, Brest, France; Neurology Department, Rennes University Hospital, Rennes, France

**Keywords:** structural connectome, tractography, brain aging

## Abstract

Diffusion-weighted MRI samples the directional diffusion of water in vivo, and tractography uses this information to reconstruct brain fiber pathways. Mapping streamlines to an anatomical parcellation yields structural connectomes. However, in older adults, where white matter alterations and atrophy are common, the choice of reference template and tissue segmentation could be particularly consequential for obtaining accurate, interpretable structural connectomes. Using a conventional pipeline, we evaluated how these two factors, the normalization template and the segmentation used to constrain anatomically guided tractography, affect connectome estimates in cognitively healthy elderly participants. These methodological choices produced systematic and significant differences in network topology and their relationships with clinically relevant variables across standard connectome measures, particularly in patients with white matter hyperintensities. Age-appropriate normalization and lesion-aware anatomically constrained tractography yielded networks with more plausible anatomy and more consistent relationships with cognition and imaging measures, whereas unconstrained tracking inflated density without improving interpretability. These findings demonstrate that structural connectome outcomes are contingent upon template and segmentation selection. We, therefore, advocate for explicit reporting of these methodological parameters and recommend the use of age-appropriate templates combined with white matter lesion-aware segmentation incorporating anatomically informed constraints.

## 1 Introduction

Diffusion-weighted imaging (DWI) infers the orientation of water diffusion within brain tissues, enabling in-vivo characterization of white matter (WM) microstructure (Alexander et al., 2019). Exploiting the anisotropic water diffusion in WM, tractography algorithms reconstruct trajectories that approximate WM architecture, offering a mesoscale scaffold on which to study brain organization (Jeurissen et al., 2019; Takemura et al., 2024).

Despite substantial progress, community challenges and systematic reviews show that current tractography algorithm outcomes remain highly sensitive to sequence acquisition parameters, diffusion models, and algorithm choices (Schilling et al., 2021), with both false positives and false negatives remaining key concerns (Maier-Hein et al., 2017; Schilling et al., 2019; Thomas et al., 2014). For better controlling false positive connections, complementary approaches have been proposed, including signal-consistency filtering (Daducci et al., 2015; Smith et al., 2015), improved orientation modeling (Jeurissen et al., 2014), and bundle-specific priors (Durantel et al., 2025; Rheault et al., 2019). Among these, anatomically constrained tractography (ACT) leverages tissue segmentation to enforce biologically plausible seeding and termination rules (Smith et al., 2012). The ACT algorithm has become widely adopted in large scale studies (Mansour et al., 2023; Wainberg et al., 2024) and recommended in modern pipelines (Tahedl et al., 2025). Empirically, ACT increases anatomical plausibility relative to unconstrained tracking (Yeh et al., 2016) and has shown good performance in recent community challenges (Girard et al., 2023), supporting current recommendations for its use in tractography (F. Zhang et al., 2022).

Structural connectomes are obtained by mapping WM pathways to an anatomical atlas so that regions become nodes and streamlines become edges, yielding undirected graphs on which global and nodal topological properties can be computed (Sotiropoulos & Zalesky, 2019; Yeh et al., 2021). These graph measures offer a compact summary of brain organization and allow comparisons across cohorts (Meskaldji et al., 2013). However, both global and nodal properties are sensitive to methodological choices in connectome definition and construction (Rubinov & Sporns, 2010; Wijk et al., 2010); this warrants explicit reporting and careful interpretation.

The study of structural connectomics is particularly valuable in aging research, where subtle WM integrity changes can facilitate the differentiation between older adults who will undergo pathological cognitive decline and those who will not, for example in the context of mild cognitive impairment, but also prior to the emergence of clinically detectable impairment (Berlot et al., 2016; Peraza et al., 2019). For instance, lower efficiency and longer paths have been related to worse cognitive performance in older adults and prodromal disease (Boot et al., 2020; Fischer et al., 2021; Lawrence et al., 2014). A converging literature also links cerebrovascular burden, most commonly indexed by the load of white matter hyperintensities (WMH), to connectivity disruption (Wardlaw et al., 2015). In late life, WMH are widespread and have been linked with reduced structural network efficiency and altered topology, providing a plausible pathway to cognitive decline (Cai et al., 2021; Rudolph et al., 2024; Taghvaei et al., 2024; Yang et al., 2020). Crucially, however, these topological measures are highly sensitive to choices made during network modeling (De Reus & Van Den Heuvel, 2013).

Connectome reconstruction relies heavily on the anatomical accuracy of tissue segmentation to define streamline start and stop rules. This is particularly critical for ACT, where mislabeling tissue types directly alters streamline trajectories and terminations. In aging brains, standard intensity-based segmentation tools such as FSL FAST (Y. Zhang et al., 2001) often misclassify WMH as gray matter (GM). This error causes ACT to prematurely terminate streamlines at lesion sites, potentially creating artificial network disconnections (Theaud et al., 2017). Conversely, methods incorporating probabilistic tissue priors, such as ANTs Atropos (Avants, Tustison, Wu, et al., 2011), may stabilize segmentation in these heterogeneous regions, thereby preserving the continuity of white matter tracts involved in pathological processes. In addition, methods based on convolutional neural networks (CNN), such as FreeSurfer WMH-SynthSeg (Laso et al., 2024), offer a lesion-aware strategy that explicitly models WMH during tissue segmentation. By treating WMH as a distinct class, WMH-SynthSeg reduces lesion-related misclassification and provides a more faithful tissue delineation. Parallel to segmentation challenges, the choice of reference template for spatial normalization introduces another source of bias. While the young adult Montreal Neurological Institute (MNI) template (Fonov et al., 2009) remains ubiquitous, age-specific templates can reduce registration bias by better capturing cortical thinning, ventricular enlargement, and other features of older brains (Fillmore et al., 2015). The MIITRA project (Ridwan et al., 2021) provides high-resolution T1-weighted (T1w) and diffusion-tensor templates specifically for non-demented older adults, with evidence of improved anatomical representativeness and sharpness relative to general templates. In summary, we hypothesize that the combined use of intensity-based segmentation and standard, young-adult templates fails to account for the specific morphology of the aging brain, leading to systematic artifacts in structural connectivity. Therefore, adopting lesion-aware segmentation strategies and age-appropriate templates is essential to recover accurate and biologically plausible network topologies. In this study, we systematically evaluate how tissue segmentation (FSL FAST, ANTs Atropos, and FreeSurfer WMH-SynthSeg) and the reference template (MIITRA and MNI) used for spatial normalization affect structural connectome estimates in cognitively normal older adults. Analytically, we proceed in three steps: (i) within each pipeline, we quantify template effects on global network measures; (ii) we test whether the relationships between structural connectome properties and four clinically relevant variables related to imaging and cognitive factors differ among pipelines; and (iii) we assess template effects on nodal measures, summarizing spatial coverage and network-level patterns. Taken together, this design aims to assess how template selection and segmentation choices propagate to graph topology and their relationships with clinically relevant variables in a sample of healthy elderly participants.

## 2 Materials and Methods

### 2.1 Data

Data were obtained from the Alzheimer’s Disease Neuroimaging Initiative^1^ (ADNI), with only participants from the ADNI-3 release (Weiner et al., 2017) included in this study.

#### 2.1.1 Included participants

Fifty cognitively normal (CN) older adults were included. Inclusion criteria required the availability of both advanced DWI protocol and high-resolution T1w MRI acquired on the same visit. Moreover, inclusion was restricted to participants with a multi-shell diffusion acquisition, which enables the use of multi-fiber and multi-tissue models, improving fiber orientation estimation and tractography accuracy (Jeurissen et al., 2014). In addition to imaging, we retained from the dataset the following imaging and cognitive measures: amyloid-β Centiloid (Aβ-Cent), which quantifies cortical amyloid load on a standardized scale where higher values indicate greater amyloid deposition (Iaccarino et al., 2025); white matter hyperintensity burden (WMHB), defined as WMH volume relative to total WM volume, with higher values indicating more extensive lesions (Wardlaw et al., 2013); and two global cognitive screening tests, the Mini Mental State Examination (MMSE) (Folstein et al., 1975) and the Montreal Cognitive Assessment (MoCA) (Nasreddine et al., 2005), both ranging from 0 to 30, with higher scores denoting better cognitive functioning. All participants provided written informed consent, and the ADNI study was approved by the institutional review boards of all participating sites. Information for the included participants is reported in Table 1.

**Table 1.** Characteristics of the included participants.

Variable	Value	Range	Unit
Age	75.62±7.28	65 – 92	Years
Sex (F/M)	27/23	–	–
Education	16.76±2.29	11 – 20	Years
Aβ-Cent	21.42±33.44	−22 – 108	–
WMHB	1.54±3.10	0.01 – 19.57	%
MMSE	29.04±1.23	25 – 30	–
MoCA	25.88±2.38	20 – 30	–

#### 2.1.2 MRI procedure

MRI scans were acquired across 11 acquisition sites using Siemens 3T Prisma and Prisma fit systems. The protocol included high-resolution T1w structural imaging and multishell DWI. Structural scans consisted of a sagittal T1w magnetization-prepared rapid gradient-echo (MPRAGE) sequence (TR = 2300 ms, TE = 2.98 ms, TI = 900 ms, flip angle = 9°, voxel size = 1.0×1.0×1.0 mm). Diffusion images were acquired using a multiband-accelerated (factor 3) monopolar spin-echo EPI sequence (TR = 3400 ms, TE = 71 ms, flip angle = 90°, voxel size = 2.0×2.0×2.0 mm). The protocol included three diffusion shells (b = 500, 1000, and 2000 s/mm^2^) for a total of 112 gradient directions.

### 2.2 Image preprocessing

All images were preprocessed using a combination of the Anima toolbox^2^, the FMRIB Software Library (FSL v6.0.7.17, Jenkinson et al., 2012), the Advanced Normalization Tools (ANTs v2.6.0.dev1-gb775a15, Avants et al., 2008), and MRtrix3 (v3.0.5, J.-D. Tournier et al., 2019) software packages.

#### 2.2.1 Anatomical MRI preprocessing

T1w structural images were first corrected for intensity non-uniformities using the N4 bias field correction algorithm (Tustison et al., 2010), as implemented in ANTs. Brain extraction was subsequently performed using an atlas registration-based method implemented in Anima toolbox and the resulting skull-stripped images were registered to DWI volumes using FSL’s flirt. Non-linear registration to the brain-extracted structural template space (MNI or MIITRA) was performed using the antsRegistration tool from ANTs (Avants, Tustison, Song, et al., 2011), following a multi-stage procedure. The pipeline included an initial rigid alignment (Rigid[0.1]) and an affine transformation (Affine[0.1]), both optimized using mutual information (MI) as the similarity metric with 32 bins and a regular sampling of 25%. Finally, a non-linear registration was carried out using the SyN transformation model (SyN[0.1, 3, 0]) with cross-correlation (CC, radius = 4) as the similarity metric. Each stage was iteratively optimized with four resolution levels (-f 8 x 4 x 2 x 1) and Gaussian smoothing (-s 3 x 2 x 1 x 0vox). The convergence criterion was defined as 10^−7^ with up to 1000 iterations per level for the affine stages and 200 iterations per level for the SyN stage. Forward and inverse deformation fields were generated and retained for subsequent application in connectome construction.

#### 2.2.2 Diffusion MRI preprocessing

Diffusion images underwent a comprehensive preprocessing pipeline designed to correct for acquisition-related artifacts and improve signal quality. Denoising was performed using Marchenko–Pastur principal component analysis (MP-PCA) implemented in MRtrix3, which jointly estimates the local noise level and removes Gaussian noise components from the diffusion data, thereby improving signal-to-noise ratio while preserving fine anatomical detail (Veraart et al., 2016). Initial correction for head motion and eddy current-induced distortions was performed using FSL’s eddy tool. Susceptibility-induced distortions were addressed using FSL’s fugue tool, based on fieldmaps acquired during the scanning session. These fieldmaps enabled estimation of the off-resonance field and generation of spatial deformation fields to correct for geometric distortions in the diffusion volumes. Subsequently, bias field correction was applied to remove residual low-frequency intensity inhomogeneities using the dwibiascorrect function from MRtrix3, which internally leverages the N4 algorithm implemented in ANTs (Tustison et al., 2010). This step improves intensity homogeneity across the brain and supports more accurate modeling of white matter microstructure. Finally, brain extraction was performed by applying the brain mask derived from the preprocessed T1w structural image. All preprocessing steps were applied identically across all experimental pipelines to ensure consistency and allow downstream methodological variations to be isolated and systematically evaluated.

### 2.3 Tractography pipeline

Whole-brain tractography was performed using MRtrix3. Tissue-specific response functions for WM, GM, and cerebrospinal fluid (CSF) were computed using the dwi2response dhollander algorithm (Dhollander et al., 2016), and the corresponding fiber orientation distributions (FODs) were calculated using dwi2fod msmt_csd (Jeurissen et al., 2014). All computations were performed within the brain mask and using a maximum spherical harmonic order of $l_{\text{max}}\text{​}=8$ for all compartments. Tractography was performed with tckgen using the iFOD2 probabilistic algorithm (J. D. Tournier et al., 2010) and the default parameters for maximum streamline length and angular threshold. For pipelines incorporating ACT, streamlines were seeded from the gray matter–white matter interface, and anatomical tissue segmentation was provided via a five-tissue-type (5TT) image. In contrast, for the pipeline excluding ACT, the brain mask was used as the seed region, given the absence of tissue segmentation in these pipelines. A fixed number of 10 million streamlines were generated with dynamic seeding and backtracking enabled to improve coverage and anatomical plausibility. Streamline weights were subsequently refined using SIFT2 (Smith et al., 2015), via the tcksift2 command, which adjusts each streamline’s contribution based on the underlying FOD field. This step enhances the quantitative accuracy of structural connectivity estimates by aligning streamline counts more closely with local fiber density.

### 2.4 Connectome construction and graph-theoretical analysis

For each subject, atlas labels from the Schaefer 400-region (Schaefer et al., 2018) and the Tian 16-region (S1 scale) (https://doi.org/10.1038/s41593-020-00711-6) parcellations were warped from the corresponding reference template (MNI or MIITRA) space to subject space using the inverse deformation field derived from the T1w-to-template registration described in Section 2.2.1.^3^ Connectivity matrices were generated with tck2connectome from MRtrix3, which maps SIFT2-weighted streamlines to anatomical regions from the combined parcellation. The resulting matrices were treated as weighted, undirected graphs: nodes correspond to 416 brain regions and edges encode anatomically informed connection strength (SIFT2-weighted streamline counts). For network level annotation, cortical nodes were assigned to the Yeo-7 functional networks (Thomas Yeo et al., 2011) and subcortical nodes retained their Tian labels.

A series of global and local network metrics were computed as described in Rubinov and Sporns, 2010, and using the NetworkX (v3.5) library in Python (v3.13.3). For path-based metrics, edge lengths were defined as the inverse of weights, so that stronger connections corresponded to shorter distances. Formal definitions of these measures are provided in the Supplementary Material (Section 1.1).

### 2.5 Experimental conditions

To systematically assess the influence of processing choices on structural connectome reconstruction, a set of pipelines was designed varying two key factors: the structural template and the tissue segmentation strategy used for ACT. An overview of the structural connectome pipeline is shown in Figure 1. Each subject’s T1w image was nonlinearly registered to template space with antsRegistration (as explained in Section 2.2.1), using two population-based templates: the widely used MNI template, specifically the MNI152NLin2009 version (Fonov et al., 2009), and MIITRA, a template specifically designed for older adults (Ridwan et al., 2021). The inverse deformation fields were retained and used to bring template-based anatomical priors into subject space when needed.

**Fig. 1.**
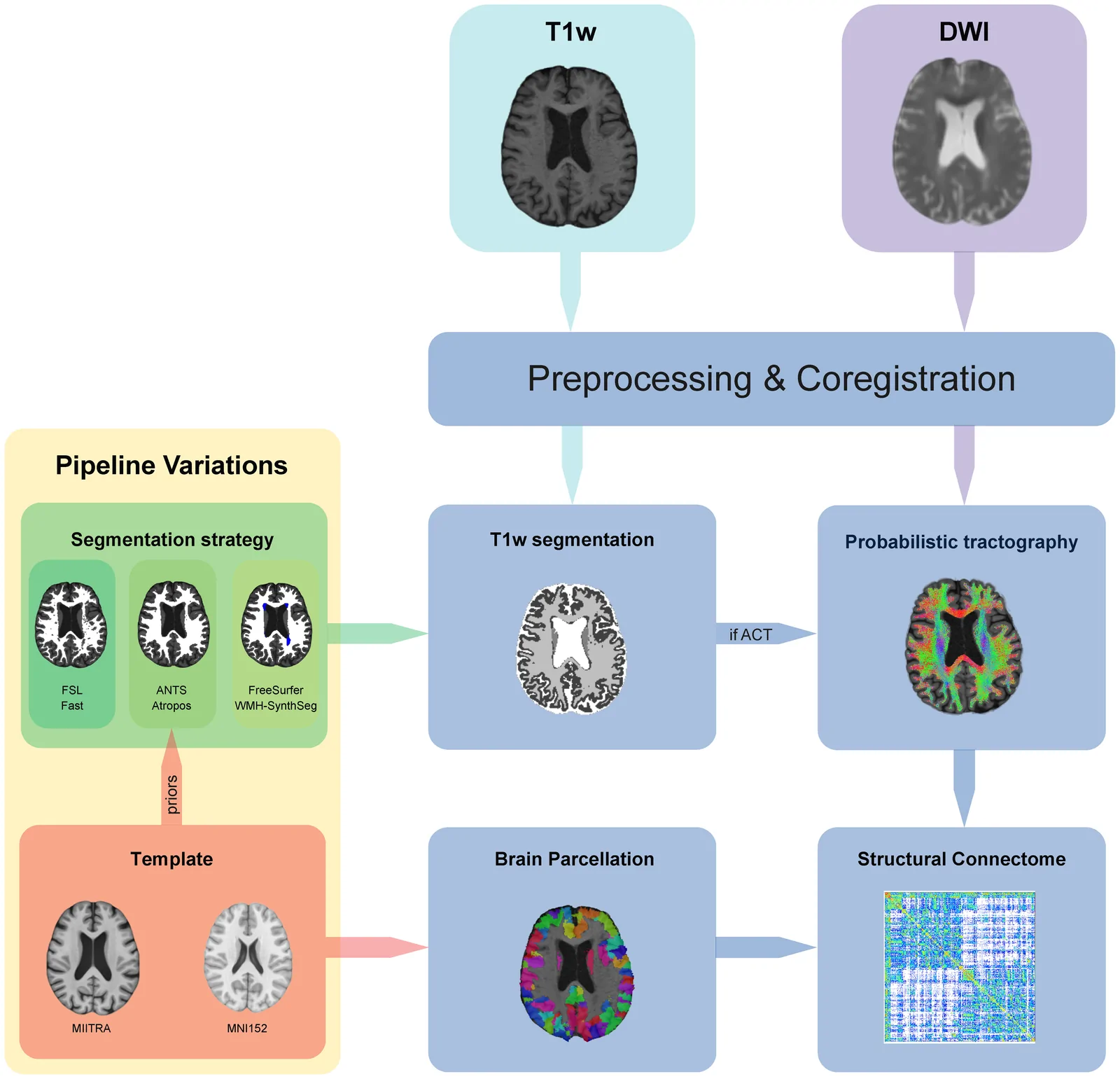
Overview of the structural connectome reconstruction pipeline. T1w and DWI images undergo preprocessing and coregistration, followed by T1w segmentation and brain parcellation; whole-brain probabilistic tractography is then performed with or without ACT, and streamlines are aggregated into the structural connectome. The pipeline explicitly varies the template (MIITRA or MNI) and the segmentation strategy (FSL FAST, ANTs Atropos, or FreeSurfer WMH-SynthSeg) to assess their impact on downstream connectome estimates.

Tissue segmentation for ACT was carried out using three alternative approaches. In the first case, the FAST algorithm (Y. Zhang et al., 2001) was used to generate WM, GM, and CSF probability maps directly from the T1w image. In the second case, the Atropos algorithm from ANTs was employed (Avants, Tustison, Wu, et al., 2011), which incorporates probabilistic tissue priors warped from template space into subject space. The corresponding white matter priors for the MNI and MIITRA templates are shown in Supplementary Figure S1. To evaluate the sensitivity of segmentation to prior information, Atropos was run with three different prior weights: 0.20, 0.35, and 0.50. In the third case, the WMH-SynthSeg algorithm was used (Laso et al., 2024), a lesion-aware segmentation approach based on a CNN that segments WMH together with the main tissue classes in a unified framework. For the FAST- and Atropos-based pipelines, subcortical structures, including the thalamus, caudate, putamen, globus pallidus, hippocampus^4^, and amygdala, were segmented using the FSL tool run_first_all, which combines intensity profiles with Bayesian statistical shape and appearance models to delineate deep gray matter nuclei (Patenaude et al., 2011). In these pipelines, the fifth volume was then defined as the set of voxels not assigned to WM, GM, CSF, or subcortical structures. For the WMH-SynthSeg-based pipeline, subcortical structures were segmented jointly with the other tissue classes, and the fifth volume included voxels labeled as WMH. These regions and compartments were then integrated together to produce a 5TT image for use in ACT-based tractography.

These methodological choices resulted in 12 distinct pipelines, 2 templates × 6 variants. The six variants comprise one configuration using ACT with FSL FAST (FAST-ACT), three configurations using ACT with ANTs Atropos at varying prior weights (collectively, ATR–ACT), one configuration using FreeSurfer WMH-SynthSeg (WMH-ACT), and one configuration without ACT (NOACT), used as a reference. An overview of the pipelines can be found in Table 2. This experimental design allows for a systematic evaluation of how processing decisions, individually and in combination, affect the topology and the properties of derived structural connectomes. All other tractography procedures were held constant to isolate the specific impact of template selection and segmentation strategy.

**Table 2.** Overview of structural connectome reconstruction pipelines varying template and segmentation strategy.

Pipeline ID	Template	Segmentation strategy	Prior weight	ACT
MNI-FAST-ACT	MNI	FSL FAST	–	Yes
MIITRA-FAST-ACT	MIITRA	FSL FAST	–	Yes
MNI-ATR-LOW-ACT	MNI	ANTs Atropos	0.20	Yes
MIITRA-ATR-LOW-ACT	MIITRA	ANTs Atropos	0.20	Yes
MNI-ATR-MED-ACT	MNI	ANTs Atropos	0.35	Yes
MIITRA-ATR-MED-ACT	MIITRA	ANTs Atropos	0.35	Yes
MNI-ATR-HIG-ACT	MNI	ANTs Atropos	0.50	Yes
MIITRA-ATR-HIG-ACT	MIITRA	ANTs Atropos	0.50	Yes
MNI-WMH-ACT	MNI	FreeSurfer WMH-SynthSeg	–	Yes
MIITRA-WMH-ACT	MIITRA	FreeSurfer WMH-SynthSeg	–	Yes
MNI-NOACT	MNI	–	–	No
MIITRA-NOACT	MIITRA	–	–	No

### 2.6 Statistical analysis

All statistical analyses were performed in Python (v3.12.2) using scipy (v1.13.1) and statsmodels (v0.14.2). For global network measures, paired comparisons between reference templates (MIITRA vs. MNI) were conducted within each segmentation strategy. For each subject, the difference in metric values between templates was computed and normality of these paired differences was assessed using the Shapiro–Wilk test. When normality was satisfied, paired t-tests were applied; otherwise, the Wilcoxon signed-rank test was used. To account for multiple testing across all metrics and pipelines, p-values were corrected at the family level using the Benjamini–Hochberg false discovery rate (FDR) procedure. Corrected p-values (q-values) were used for visualization in boxplots, where significance was denoted by symbols corresponding to conventional thresholds (^†^q<0.05 , ^‡^q<0.01 , ^§^q<0.001 ).

Associations between global connectome metrics and cognitive or imaging measures were evaluated using linear mixed-effects models, with the measure, pipeline (template × segmentation), and their interaction used as fixed effects and subject as a random intercept. Four measures were examined: Aβ-Cent, WMHB, MMSE, and MoCA scores. Age, sex, and total cerebral volume were included as covariates to control for demographic and anatomical variability. For each measure–metric pair, the omnibus interaction test (measure × pipeline) using a Wald χ^2^ statistic is reported, as well as the estimated slopes and their 95% confidence intervals within each pipeline. Pairwise comparisons of slopes across pipelines were corrected for multiple testing using FDR, and results were summarized both in tabular form and as heatmaps of corrected q-values. To complement these model-based results, Pearson correlation coefficients were also computed separately for each pipeline as descriptive measures of association.

For nodal network metrics, the effect of template choice was assessed while accounting for differences in segmentation strategies. For each node and technique, a mixed-effects model was fitted with template, technique, and their interaction as fixed effects, including age, sex, and total cerebral volume as covariates, and subject as a random intercept. For each node–technique pair, we estimated the effect of template (MIITRA vs. MNI) and assessed its significance. The p-values across nodes were corrected using the Benjamini–Hochberg FDR procedure within each metric. Significant effects were visualized in heatmaps where nodes were ordered according to the Yeo-7 network partition, allowing network-level summaries of template-related biases. Additionally, the percentage of nodes showing significant differences in template effect between pipelines was quantified, providing an estimate of cross pipeline variability.

## 3 Results

### 3.1 WM segmentation evaluation between segmentation strategies

Figure 2 shows the comparison between WM segmentation obtained with FSL FAST, ANTs Atropos, and FreeSurfer WMH-SynthSeg in three representative subjects. FAST systematically failed to classify WMH as WM, leading to an underestimation of the true WM mask. This effect is particularly obvious in the periventricular areas, where WMH are more likely to occur and were consistently excluded from the segmented WM, thus generating false negatives (see the first line of Fig. 2). Conversely, Atropos showed a more consistent inclusion of WMH within the WM segmentation. The performance depended on the choice of the prior weighting factor δ. Lower values of δ (δ=0.20 ) resulted in misclassification of WMH voxels as GM. Increasing the prior weight improved the correct labeling of WMH as WM, reducing segmentation errors, as for δ=0.35 or δ=0.50 . WMH-SynthSeg provided the most homogeneous WM segmentation across subjects, while also explicitly identifying WMH as a separate tissue class (in blue). This lesion-aware labeling improved the continuity of the WM mask in affected regions and reduced the discontinuities observed with the other segmentation approaches. Overall, these findings indicate that lesion-aware segmentation strategies improve WM delineation in the presence of WMH, with WMH-SynthSeg yielding the most uniform WM representation and Atropos showing performance that depended on prior weighting.

**Fig. 2.**
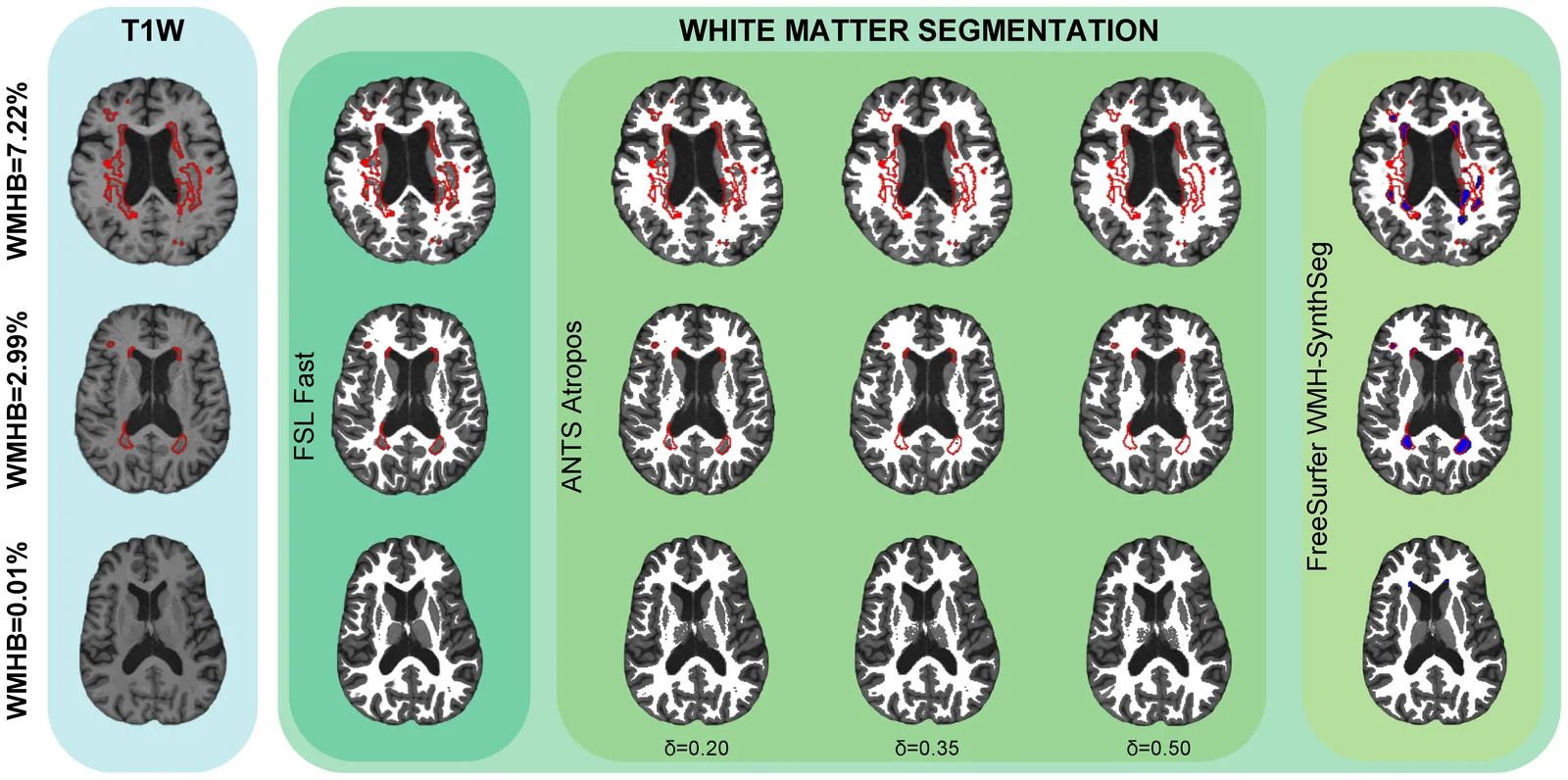
Results of white matter segmentation in three representative subjects with different WMH burdens (WMHB = 7.22%, 2.99%, and 0.01%). The first column shows the original T1w images. The second column displays the WM segmentation obtained with FSL FAST. The central columns illustrate ANTs Atropos segmentations obtained with different prior weighting factors (δ=0.20,0.35,0.50 ) using MIITRA template priors registered to subject space. The last column shows the WM segmentation obtained with WMH-SynthSeg, together with the corresponding WMH segmentation (blue). WMH contours derived from FLAIR images (red) are overlaid on all images.

### 3.2 Global network properties

In the ATR-ACT configurations, the MIITRA template shows generally higher sparsity, mean connectivity, global efficiency, and assigned streamlines than MNI, as displayed in Figure 3. The magnitude of these differences is most evident for mean connectivity and global efficiency, whereas the effect on sparsity and assigned streamlines appears less regular across Atropos prior weights. In the FAST-ACT pipeline, MIITRA tends to show lower sparsity, mean connectivity, global efficiency, and assigned streamlines than MNI. In the WMH-ACT and NOACT configurations, template-related differences follow a different pattern, with MIITRA showing higher global efficiency but lower sparsity, mean connectivity, and assigned streamlines. Results for path length and higher order descriptors are provided in the Supplementary Material (Supplementary Fig. S2 and Supplementary Table S1). Overall, the global network measures indicate that both template and segmentation strategy systematically influence network density, average connection strength, integration, and the proportion of streamlines assigned to the connectome.

**Fig. 3.**
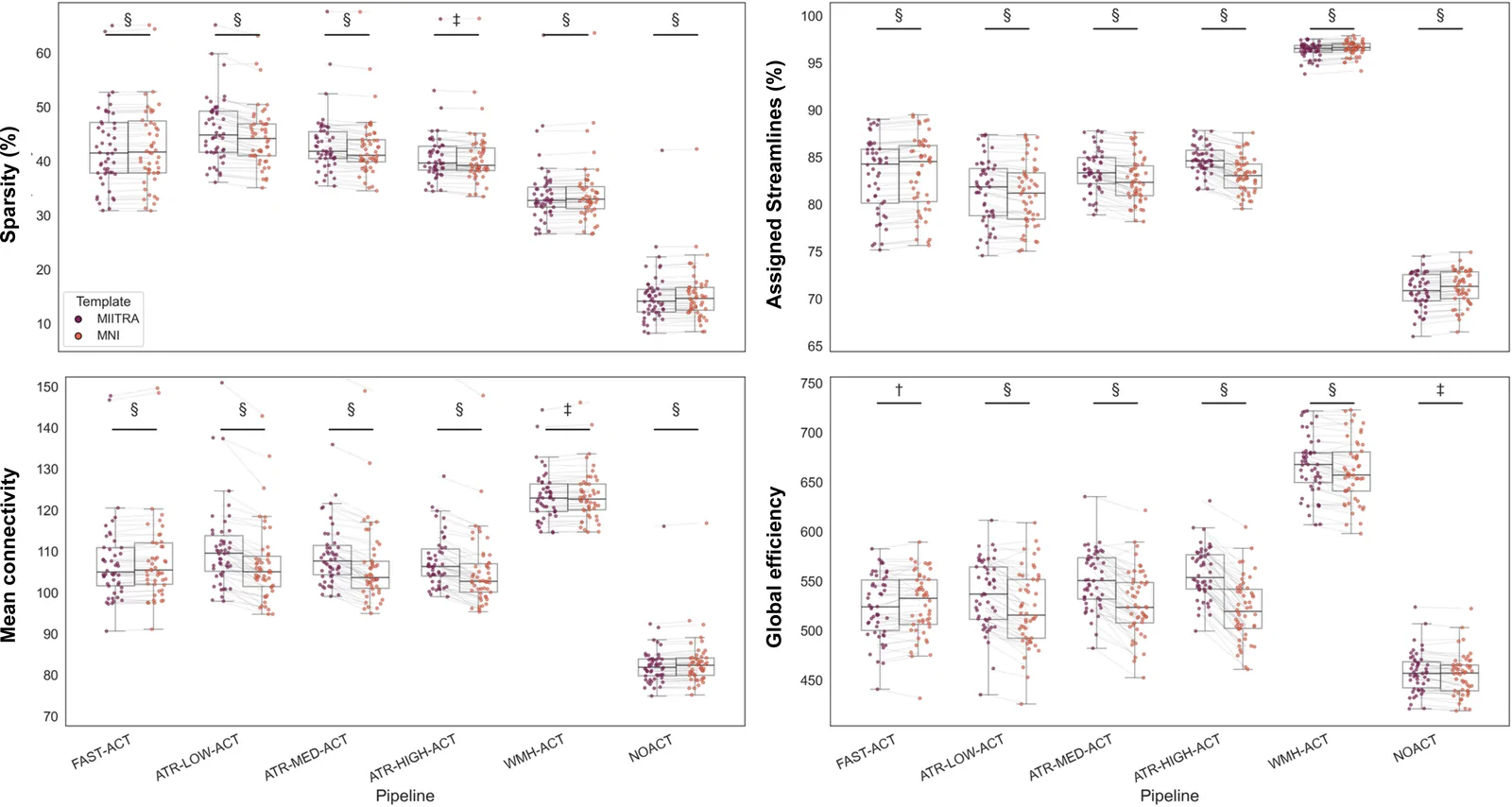
Boxplots for global connectome metrics across pipelines and registration templates. Top left: sparsity (SP), bottom left: mean connectivity (MC), top right: assigned streamlines (AST), bottom right: global efficiency (GE). Symbols denote significance levels from paired *t*-tests between templates within each pipeline (^†^q<0.05 , ^‡^q<0.01 , ^§^q<0.001 ).

### 3.3 Pipeline-dependent associations

Figure 4a shows that, within each template, sparsity and mean connectivity increase with WMHB across all pipelines. The strongest positive associations are observed in the FAST-ACT configuration, which shows the steepest fitted lines and the highest *R*^2^ values for both metrics under both MIITRA and MNI. The ATR-ACT pipelines retain the same positive direction of effect, but the magnitude of the association becomes progressively weaker from ATR-LOW-ACT to ATR-HIG-ACT, with broadly comparable patterns across templates. WMH-ACT and NOACT also show positive associations for sparsity and mean connectivity, although with smaller slopes and lower explained variance than FAST-ACT.

**Fig. 4.**
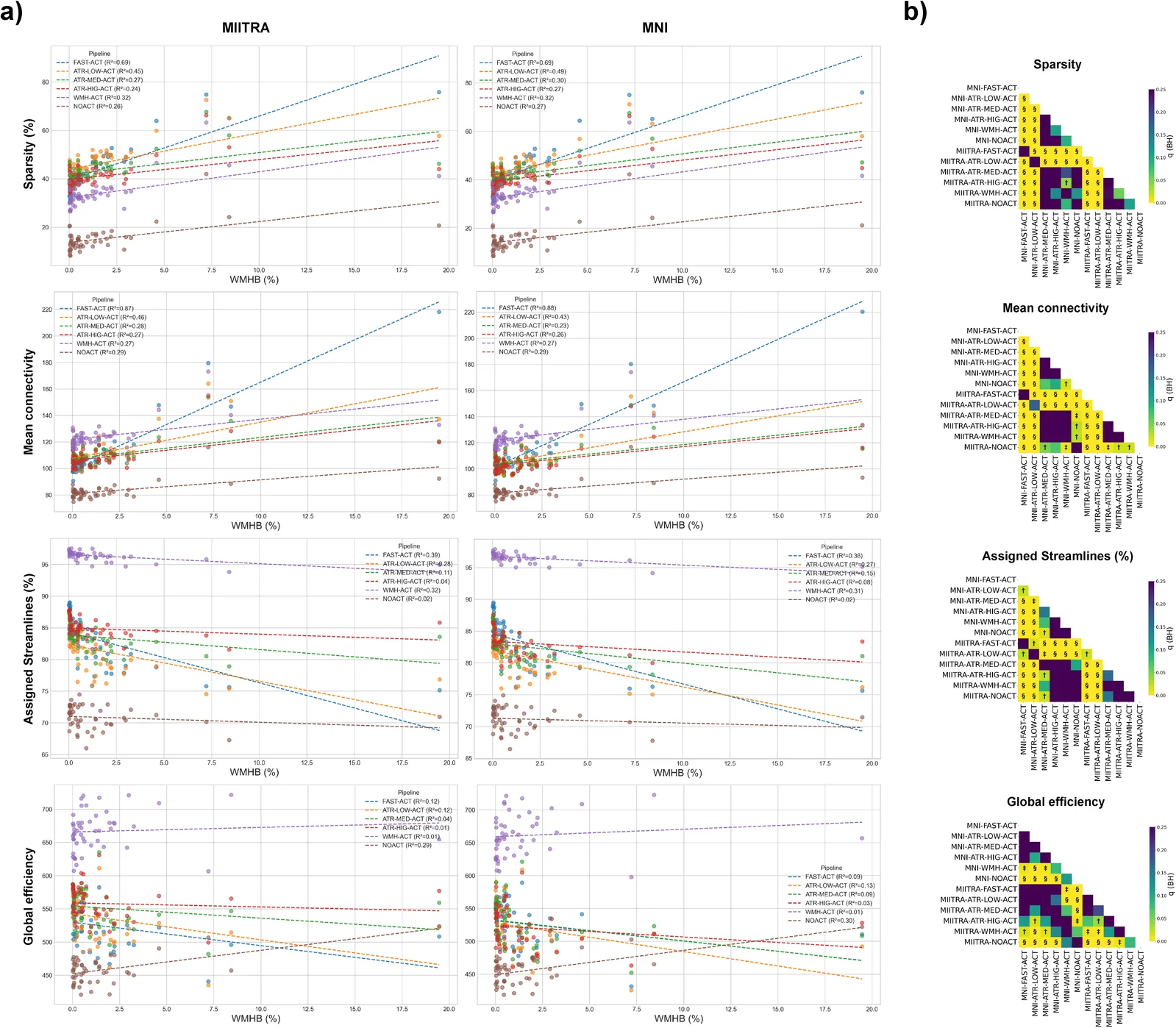
(a) Scatter plots for four global metrics (in order: sparsity (SP), mean connectivity (MC), assigned streamlines (AST), and global efficiency (GE)) shown separately by template (left: MIITRA; right: MNI) where each point is a subject (x-axis: WMHB; y-axis: metric). Dashed lines indicate per-pipeline linear fits and legends report *R*^2^ for each fit. (b) Lower-triangular heatmaps of FDR-corrected *q*-values (Benjamini–Hochberg) for pairwise differences between the slopes estimated in (a). Cells are annotated with significance markers (^†^q<0.05 , ^‡^q<0.01 , ^§^q<0.001 ).

Assigned streamlines and global efficiency show a different pattern. For assigned streamlines, the association with WMHB is negative in FAST-ACT, ATR-LOW-ACT, ATR-MED-ACT, and WMH-ACT configurations, while it becomes markedly attenuated in ATR-HIG-ACT and NOACT conditions. For global efficiency, negative slopes are observed in the FAST-ACT and ATR-LOW-ACT configurations. In contrast, the NOACT configuration shows a positive association with WMHB, whereas the remaining pipelines exhibit nearly flat relationships.

Figure 4b and Supplementary Table S3 confirm that these visual differences are statistically meaningful. For sparsity and mean connectivity, the pipeline dependence mainly reflects differences in effect size, with all pipelines preserving a positive association with WMHB. In contrast, assigned streamlines and global efficiency show clearer separation among pipelines because both the magnitude and, in some cases, the direction of the association vary across pipelines. In particular, the transition from negative to near-null or positive slopes distinguishes the WMH-ACT and NOACT configurations from FAST-ACT and the ATR-ACT pipelines.

The omnibus interaction tests (Fig. 5 and Supplementary Table S2) further indicate that the linear associations between global network properties and clinical or imaging measures depend on the pipeline. WMHB and MMSE show significant pipeline-dependent effects across all global descriptors, whereas Aβ-Cent displays widespread effects across all metrics except average clustering. By contrast, MoCA exhibits a more selective pattern, with significant interactions limited to assigned streamlines, global efficiency, and average shortest path. Overall, these results indicate that the detection and strength of associations between global network metrics and clinical or imaging measures are strongly conditioned by the connectome reconstruction pipeline, with FAST-ACT showing the strongest WMHB-related changes, the ATR-ACT pipelines showing a graded attenuation across prior weights, and WMH-ACT and NOACT producing metric-dependent patterns. Supplementary scatter plots and heatmaps are provided in Supplementary Figures S3–S6, and the Wald χ^2^ summaries in Supplementary Tables S3–S6.

**Fig. 5.**
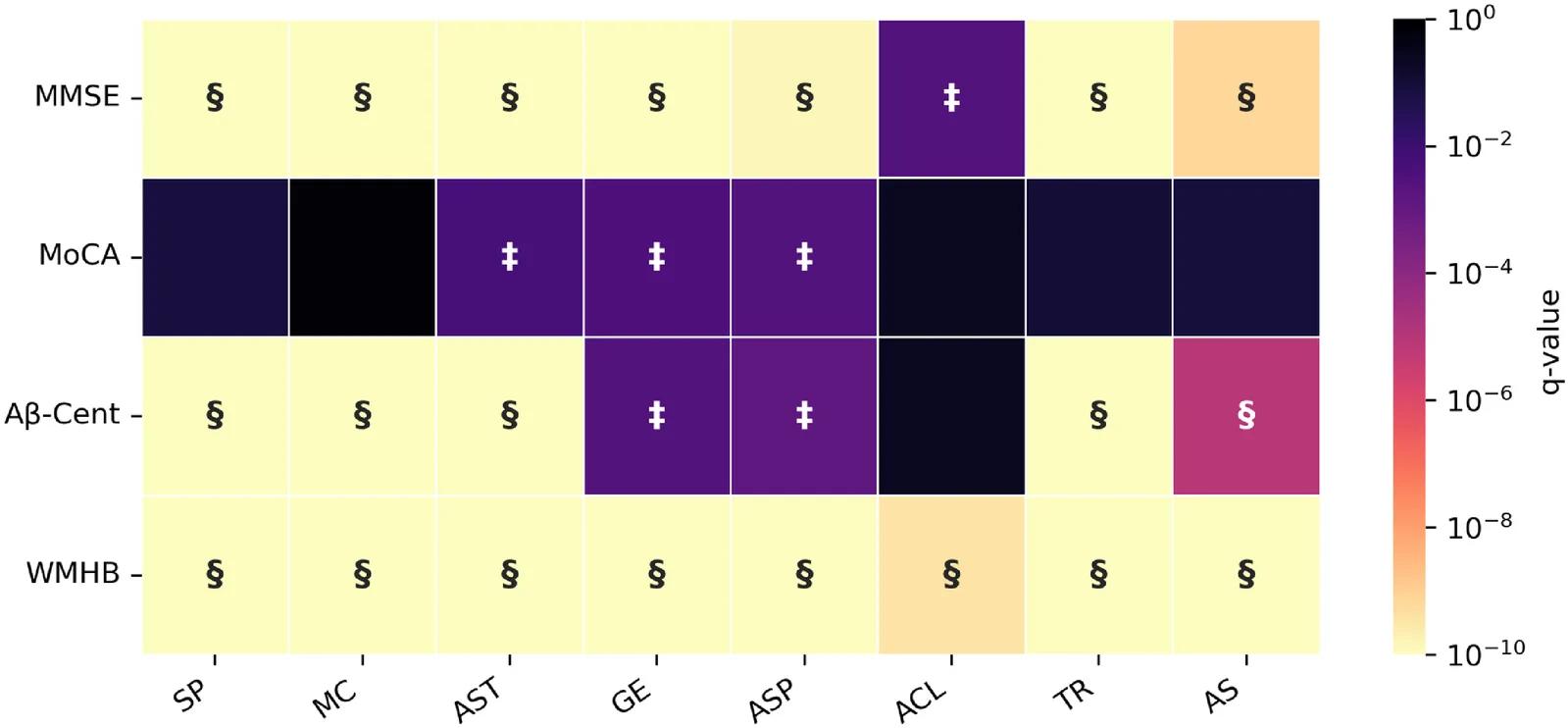
Omnibus tests (measure × pipeline) heatmap for global network metrics. Values are FDR-corrected q-values with significance levels denoted (^‡^*q* < 0.01, ^§^*q* < 0.001). SP: sparsity; MC: mean connectivity; AST: assigned streamlines, GE: global efficiency; ASP: average shortest path; ACL: average clustering; AS: assortativity; TR: transitivity.

### 3.4 Template effect on nodal network properties

Figure 6 summarizes node-wise template-related differences by pipeline and network. For strength, all pipelines show numerous nodes with significant template-related differences across cortical and subcortical networks. For local efficiency, ATR–ACT exhibits widespread significant differences, whereas FAST–ACT, WMH-ACT, and NOACT yield very few significant nodes. For clustering, significant differences occur mainly in subcortical nodes using ATR–ACT and NOACT, with fewer cortical nodes reaching significance; FAST–ACT and WMH-ACT show fewer significant nodes overall. For betweenness centrality, significant node-wise differences are sparse in all pipelines, with most effects confined to subcortical regions in ATR–ACT and WMH-ACT, more widespread effects in NOACT, and very few significant nodes, mostly in the visual network, for FAST–ACT. In ATR–ACT, the general magnitude of these node-wise effects increases with the Atropos prior weight. Overall, node-level template effects are strong and spatially extensive in ATR–ACT, whereas FAST–ACT, WMH-ACT, and NOACT produce only sparse significant differences. Further nodal results are provided in the Supplementary Material (Supplementary Fig. S7).

**Fig. 6.**
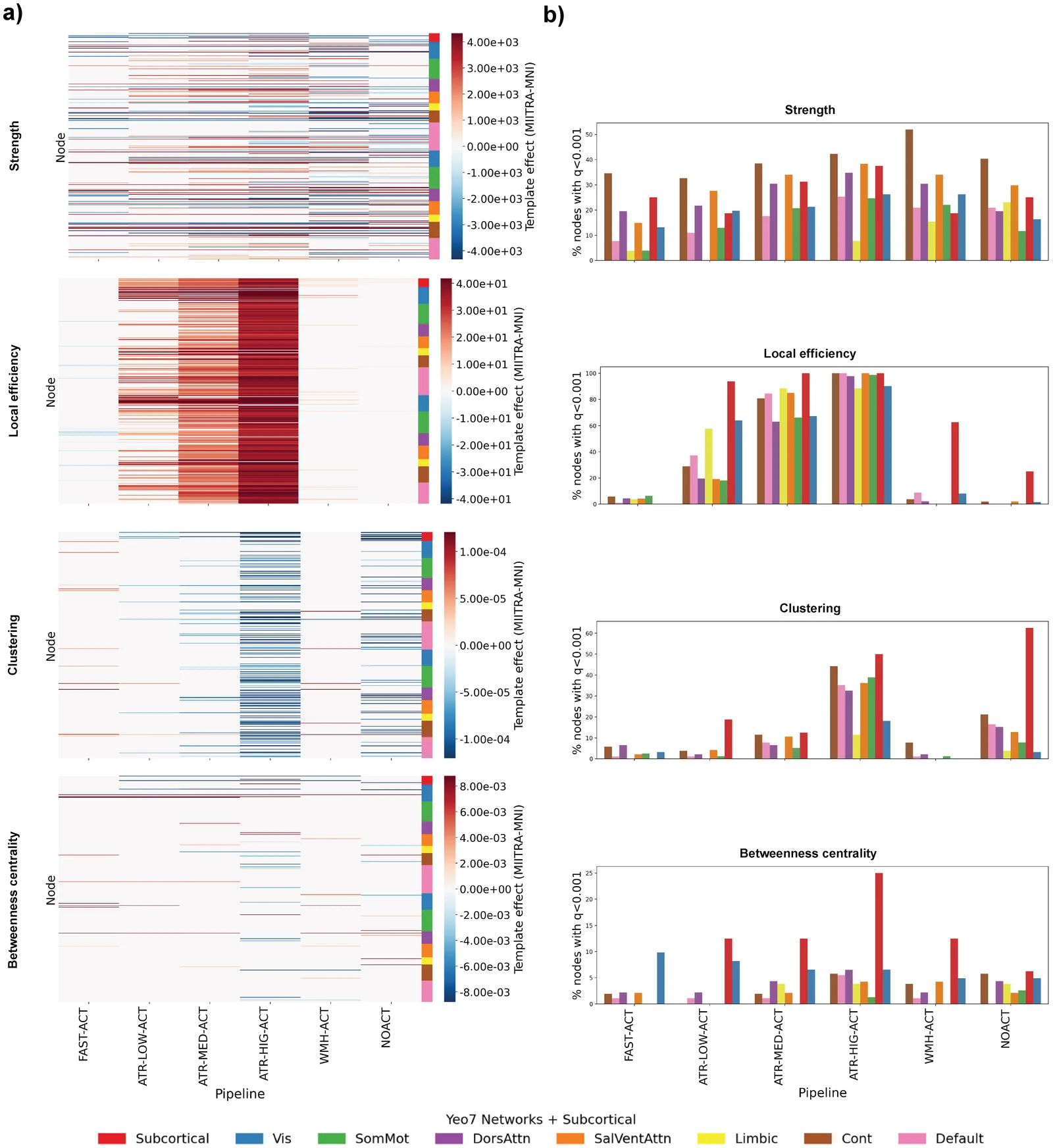
(a) Heatmaps of the MIITRA–MNI median node effect for four nodal metrics (from top to bottom: strength (ST), local efficiency (LE), clustering (CLC), betweenness centrality (BC)) across pipelines. Nodes are ordered and color coded by Yeo-7 networks plus the subcortical network (legend at the bottom). Warm/cool colors indicate positive/negative MIITRA–MNI differences. (b) For each of the four metrics shown in panel a, bar plots report the percentage of nodes with a significant MIITRA–MNI effect (q<0.001 ) across pipelines and in Yeo-7 networks.

## 4 Discussion

Reliable structural connectome reconstruction in aging brains requires accurately handling specific anatomical challenges, such as WMH and atrophy, which are often overlooked by standard pipelines. To address this, we investigated the impact of two fundamental processing choices: the tissue segmentation strategy and the reference template. Beyond quantifying differences in graph metrics, we sought to determine how these processing decisions interact to shape the topology of the reconstructed networks and whether they modulate the detection of biological associations with markers of pathology and cognitive function.

### 4.1 Impact of segmentation and template choice on global network properties

When examining global network properties, distinct pipeline-specific profiles emerged across global network properties (Fig. 3). Within the ATR-ACT configurations, sparsity remained relatively stable across prior weights, indicating that the overall density of the connectomes is only minimally affected by the exact weighting applied to tissue priors. These pipelines also showed relatively homogeneous profiles in the other global metrics, supporting the view that the Atropos framework yields structurally consistent connectomes across prior settings. FAST-ACT departed from this pattern by showing greater variability, reflecting differences in the delineation of tissue classes when segmentation is based on intensity information alone. A more distinctive profile was observed for the WMH-ACT pipeline. Compared with FAST-ACT and the ATR-ACT configurations, WMH-ACT yielded a markedly higher proportion of assigned streamlines, indicating that a larger fraction of the reconstructed tractogram was successfully incorporated into the final connectome. This was accompanied by lower sparsity, together with higher mean connectivity and higher global efficiency. In combination, these findings suggest that explicitly modeling WMH has consequences that extend beyond lesion labeling itself, promoting the reconstruction of denser and more integrated networks. By contrast, the NOACT condition, although also characterized by low sparsity, showed a clearly different global profile. In the absence of anatomical constraints, the reduction in sparsity is likely to reflect the inclusion of spurious streamlines rather than a more biologically plausible reconstruction, and this interpretation is supported by the fact that NOACT did not reproduce the same increase in assigned streamlines and global efficiency observed for WMH-ACT. Thus, similar density values can arise from qualitatively different tractography settings and should not be interpreted in isolation. In fact, this comparison should also be interpreted in light of the different seeding strategy used in NOACT, which relies on uniform brain-mask seeding rather than GM/WM interface seeding. Against this background, template-related differences were most evident in the ATR-ACT pipelines. In this setting, MIITRA-derived connectomes generally showed slightly higher sparsity and higher mean connectivity, assigned streamlines, and especially global efficiency than the corresponding MNI-derived connectomes. Because Atropos explicitly relies on template tissue priors, these differences likely arise from the use of different prior information during segmentation. In particular, the age-matched priors provided by MIITRA are likely to offer a better anatomical fit for this cohort, thereby supporting a more coherent reconstruction of the connectome and leading to systematically higher global metrics, most clearly for global efficiency. Template effects in the remaining pipelines were significant but less marked. In WMH-ACT, for example, MIITRA was generally associated with fewer assigned streamlines than MNI, but this did not translate into lower global efficiency. This dissociation suggests that, when segmentation is more lesion-aware and anatomically appropriate, differences in global efficiency may depend less on the absolute number of assigned streamlines and more on the suitability of the reference template itself. In this sense, the lower efficiency observed under MNI may reflect the use of a template that is less well matched to the anatomy of older adults.

Overall, these findings underscore that both segmentation strategy and template choice shape the global properties of the structural connectome, albeit in different ways. Segmentation primarily determines the overall profile of the reconstructed network, with WMH-ACT standing out for its combination of higher assigned streamlines, lower sparsity, and higher mean connectivity and global efficiency, whereas NOACT shows that low sparsity alone does not imply a more plausible connectome organization. Template choice, in turn, introduces additional variation, particularly for network integration, that scales with the template’s role in segmentation: it is largest in ATR-ACT, where template priors directly inform tissue segmentation, and weakest where they do not.

### 4.2 Methodological biases in the relationship between clinical factors and network properties

The omnibus interaction tests for global metrics showed that the associations between network properties and each cognitive or imaging measure are strongly pipeline dependent. Across descriptors, FAST-ACT and NOACT systematically bias these relationships in opposite directions: FAST-ACT generally yields steeper slopes and higher *R*^2^ values, giving the impression of stronger measure–network associations, whereas NOACT leads to flatter slopes and lower *R*^2^, thereby weakening or obscuring effects. ATR-ACT pipelines occupy an intermediate position, with more reproducible estimates across prior weights and templates, while WMH-ACT appears to preserve lesion-related effects without the more pronounced shifts seen in the other ACT-based strategies.

Within this general pattern, WMHB shows the most robust and widespread interactions, especially with sparsity and mean connectivity. In line with established notions that WMH disrupt long-range WM pathways, higher lesion load was associated with sparser connectomes. Importantly, however, the strength and expression of this association were highly dependent on the chosen segmentation strategy, underscoring the methodological sensitivity of connectome reconstruction in the presence of pathology. The FAST-ACT pipeline showed the steepest associations, yielding networks with both significantly increased sparsity and inflated mean connectivity in individuals with greater WMHB. This apparently paradoxical pattern can be attributed to segmentation bias: FAST tends to misclassify WMH regions as GM, thereby preventing tractography from reconstructing fibers that traverse lesion sites (Fig. 2). As a result, many potential connections are excluded, producing networks that are simultaneously “weaker,” due to reduced density, and “stronger,” due to the selective retention of high weight streamlines. Such biases risk distorting the interpretation of how vascular lesions impact network topology, as the measured associations may reflect segmentation artifacts rather than true biological effects. Studies on WMH consistently show that lesion voxels with atypical intensities relate to partially preserved WM (Min et al., 2021; Svärd et al., 2017; Wardlaw et al., 2015). For this reason, ACT pipelines must handle WMH explicitly (Theaud et al., 2017). In contrast, NOACT pipelines yielded artificially dense connectomes that were largely insensitive to WMHB. In the absence of anatomical priors, tractography is free to generate spurious streamlines, effectively masking the true impact of pathology on network density and connectivity strength. While this approach reduces the biases associated with FAST-based WM segmentations, it introduces its own form of distortion by minimizing lesion-related effects. ATR-ACT and WMH-ACT pipelines occupied an intermediate and more biologically plausible position. They preserved the expected positive association between WMHB and network sparsity, while avoiding the exaggeration introduced by FAST-ACT and the artificial density of NOACT. The association between WMHB and assigned streamlines is also particularly informative. In FAST-ACT, assigned streamlines decrease most steeply as WMHB increases, consistent with premature termination of streamlines in regions where WMH are misclassified as GM. WMH-ACT shows a similar though less steep association, but the underlying mechanism is different: by explicitly labeling WMH, the segmentation preserves lesion-affected white matter as a distinct tissue class, so the reduction more likely reflects the reduced diffusivity of fibers within lesioned tissue than a segmentation artifact. The ATR-ACT pipelines occupy an intermediate position between these two mechanisms, with a decline in assigned streamlines that is more moderate and varies with the prior weight used during segmentation. Lower prior weights tend to preserve less lesioned tissue in the WM class, whereas higher weights provide stronger anatomical constraint and partly reduce the impact of WMHB on streamline assignment. This suggests that Atropos captures both a degree of lesion-related sensitivity and a degree of prior-driven stabilization, placing it between the more artifact-prone FAST-ACT configuration and the lesion-aware WMH-ACT approach. By contrast, NOACT remains largely insensitive to lesion burden. Meanwhile, global efficiency shows a more nuanced pattern. FAST-ACT and ATR-LOW-ACT display the strongest negative associations with WMHB, while the other ACT pipelines are comparatively flatter. By contrast, NOACT shows a counterintuitive positive association with WMHB, despite its low anatomical specificity. This suggests that higher lesion burden does not necessarily reduce global efficiency in an unconstrained tractography setting, and that the apparent increase may reflect the generation and retention of spurious connections rather than a biologically meaningful improvement in network integration.

Consistent, though generally weaker, associations also emerged for global cognition and amyloid burden. MMSE and Aβ-Cent show broad pipeline-dependent effects across several global properties, with FAST-ACT and NOACT again tending to either overestimate or dampen associations relative to the more stable ATR-ACT and WMH-ACT pipelines. MoCA, in contrast, showed fewer and less consistent interactions. Given the limited variability and redundancy between MoCA and MMSE scores in this cognitively normal cohort, these associations are modest and should be interpreted with caution.

Taken together, these results indicate that the apparent impact of WMHB and other clinical factors on global network properties cannot be interpreted without explicit consideration of the connectome reconstruction pipeline. Methodological choices not only modulate the strength of observed associations but may also qualitatively alter their direction and meaning. In this context, lesion-aware segmentation strategies appear to provide a more balanced reconstruction framework in aging cohorts, where WMH are common and potentially confounding. In particular, WMH-ACT and ATR-ACT suggest that explicit handling of lesion burden can preserve biologically plausible relationships between WMHB and network topology, whereas FAST-ACT and NOACT may either amplify segmentation-related bias or obscure lesion effects altogether.

### 4.3 Template-dependent bias in nodal measures

Template choice influenced the extent and spatial distribution of nodal graph differences, indicating that local network measures are not uniformly robust to anatomical alignment. The effect was more apparent in regions where anatomical variability and registration uncertainty are greater, with template-related differences emerging more consistently in these areas.

Within the ATR-ACT pipelines, the extent of these template-driven differences depended on the prior weighting factor. Higher prior weights were associated with broader and more spatially extensive nodal differences, indicating that stronger reliance on atlas-derived information increases sensitivity to template-specific anatomical features. As prior weighting decreased, the number of affected nodes was reduced, suggesting that greater contribution from subject-specific anatomy mitigates template-driven bias. Across the other pipelines, the differences between templates were generally less pronounced, suggesting a lower sensitivity of these reconstruction strategies to template choice. Even in these cases, however, the remaining differences were not uniformly distributed across the brain but were relatively more frequent in subcortical regions. The origin of this pattern remains unclear and could reflect registration or other template-specific properties.

Overall, these findings indicate that template choice can meaningfully affect nodal graph measures, but the magnitude and spatial pattern of this influence depend on the pipeline. The stronger effects observed in ATR-ACT highlight the role of prior weighting in shaping template sensitivity, whereas the more limited differences in the other pipelines suggest greater robustness when segmentation is less constrained by template priors.

### 4.4 Limitations and perspectives

This study has some limitations that should be acknowledged. First, the absence of a ground truth connectome limits the ability to determine which segmentation strategy or template provides the most accurate representation of structural connectivity. Our conclusions are, therefore, based on relative comparisons between pipelines, rather than on an absolute benchmark of biological validity. Second, our analyses were restricted to a relatively small cohort of healthy older adults. Although this sample is highly relevant for studying age-related changes in network organization and the impact of WMH, the modest sample size limits statistical power and may reduce the generalizability of the findings to younger populations or to clinical groups with different neuropathological profiles. Third, the large number of statistical comparisons required by the study design, given the evaluation of multiple pipelines, global metrics, and nodal measures, should be considered when interpreting the results. Even with FDR correction applied within predefined families of tests, the extensive comparison space may increase the risk of chance findings, particularly at the nodal level, and, therefore, warrants cautious interpretation of isolated significant effects. Despite these limitations, the present work provides important perspectives for future research. The systematic evaluation of segmentation and template choices highlights methodological factors that can deeply shape both global and nodal network properties. Building on these findings, future studies should incorporate test–retest datasets and multi-cohort comparisons to establish the reproducibility of connectome metrics under different preprocessing strategies. In addition, dedicated FLAIR-based WMH segmentation workflows could be integrated to assess whether improved lesion detection further enhances connectome reconstruction in aging and clinical populations with high WMHB. Moreover, extending the analysis to patients with neurodegenerative or cerebrovascular conditions will be essential to determine whether the methodological sensitivities observed here generalize to pathological brains, or whether additional adaptations are needed. Finally, the development and validation of harmonization strategies that explicitly account for template and segmentation effects may help ensure comparability across studies, thereby facilitating multi-site investigations of structural connectivity in aging.

## 5 Conclusion

This study presents a systematic evaluation of structural connectome pipelines in cognitively normal older adults, isolating the effects of reference template selection and tissue segmentation while holding tractography and parcellation constant. This work provides a principled framework for understanding how preprocessing choices propagate into downstream network measures and clinical connectome analyses and the results provide an empirical basis for comparing, and potentially harmonizing structural connectome findings across pipelines. The present work shows that template and segmentation choices can substantially shape both the magnitude and interpretation of network effects in aging cohorts, with important implications for cross-study comparability. Segmentation strategy primarily affects streamline seeding and termination, thereby shaping connectome properties, whereas template choice matters mainly when it informs tissue segmentation through priors, with limited impact otherwise. Future efforts should extend this benchmarking to pathological populations and develop harmonization strategies that explicitly model template and segmentation effects. By embedding these considerations into study design and reporting, researchers can improve the interpretability, reproducibility, and translational value of structural connectomics in aging.

## Supplementary Material

Supplementary Material

## Data Availability

The dataset used in this study is available at https://ida.loni.usc.edu and described in Weiner et al. (2017). The code developed is openly accessible at https://github.com/CarloFerritto/Structural_connectomes_in_Aging

## Footnotes

1 The ADNI was launched in 2003 as a public-private partnership, led by Principal Investigator Michael W. Weiner, MD. The primary goal of ADNI has been to test whether serial magnetic resonance imaging (MRI), positron emission tomography (PET), other biological markers, and clinical and neuropsychological assessment can be combined to measure the progression of mild cognitive impairment (MCI) and early Alzheimer’s disease (AD).

2
https://github.com/Inria-Empenn/Anima-Public

3 Both parcellations are defined in MNI space; when using the MIITRA template, the transformation provided by the MIITRA project was used to map the atlas labels from MNI to MIITRA space before warping them to subject space.

4 The hippocampus is a cortical structure, but we classified it as ’subcortical’ following common brain atlas terminology.
