# Supplementary material for: Mind the brain age: How segmentation and template selection reshape structural connectomes
Source: Imaging Neurosci (Camb). 2026 Sep 30;4:IMAG.a.1384. doi: 10.1162/IMAG.a.1384 (PMC13628500; doi:10.1162/IMAG.a.1384)
Supplement: Supplementary file 1 [file IMAG.a.1384_supp.pdf]

Supplementary Material

1 Methods

1.1 Graph-theoretical metrics

Connectivity matrices were treated as weighted, undirected graphs $G = (V, E)$, where nodes V corresponded to brain regions and edges E to streamline-based connections with weights $w_{ij} > 0$ (streamline counts). All shortest-path-based metrics were computed using $1/w_{ij}$ as edge lengths.

1.1.1 Global metrics

- **Sparsity (SP):** the percentage of absent connections in the connectivity matrix:

$$\mathsf{SP} = 100 \times (1 - \frac{2|E|}{N(N-1)}),$$

  where $|E|$ is the number of non-zero entries in the connectivity matrix.

- **Mean connectivity (MC):** the average weight of all non-zero edges:

$$\mathsf{MC} = \frac{1}{|E|} \sum_{(i,j)\in E} w_{ij}.$$

- **Assigned Streamlines (AST):** the percentage of reconstructed streamlines that were successfully assigned to the connectome, computed as

$$\mathsf{AST} = 100 \times \frac{S_{\text{assigned}}}{S_{\text{total}}},$$

  where S_{assigned} is the number of streamlines assigned to at least one brain region, and S_{total} (10 millions) is the total number of reconstructed streamlines.

- **Global efficiency (GE):** the average inverse shortest path length between all pairs of nodes:

$$\mathsf{GE} = \frac{1}{N(N-1)} \sum_{i\neq j} \frac{1}{d_{ij}},$$

  where d_{ij} is the shortest path length between nodes i and j computed using $1/w_{ij}$ as edge lengths.

- **Average shortest path (ASP):** the mean shortest distance between all node pairs:

$$\text{ASP} = \frac{1}{N(N-1)} \sum_{i \neq j} d_{ij}.$$

- **Transitivity (TR):** a measure of global clustering, computed as:

$$\text{TR} = \frac{3 \times \text{number of triangles}}{\text{number of connected triples}}.$$

- **Assortativity (AS):** the Pearson correlation coefficient between the degrees of connected nodes, indicating the tendency of nodes to connect with others of similar degree.

- **Average clustering (ACL):** the mean of the local clustering coefficients (CLC), incorporating edge weights.

1.1.2 Local metrics

- **Degree (DE):** number of directly connected neighbors:

$$\text{DE}_i = \sum_j \delta(w_{ij} > 0).$$

- **Strength (ST):** total weight of edges connected to a node:

$$\text{ST}_i = \sum_j w_{ij}.$$

- **Local efficiency (LE):** efficiency of information transfer in a node's local neighborhood:

$$\text{LE}_i = \frac{1}{k_i(k_i-1)} \sum_{\substack{j,k \in \mathcal{N}(i) \\ j \neq k}} \frac{1}{d_{jk}},$$

  where $\mathcal{N}(i)$ is the set of neighbors of node i and d_{jk} are shortest path lengths computed with inverse weights.

- **Clustering coefficient (CLC):** the fraction of closed triplets around a node, weighted by the geometric mean of edge weights.

- **Betweenness centrality (CB):** the fraction of all shortest paths that pass through a node:

$$\mathsf{BC}_i = \sum_{s \neq i \neq t} \frac{\sigma_{st}(i)}{\sigma_{st}},$$

  where σ_{st} is the number of shortest paths from node s to node t, and $\sigma_{st}(i)$ is the number of those paths that pass through node i.

- **Closeness centrality (CC):** the reciprocal of the average shortest distance from a node to all others:

$$\mathsf{CC}_i = \frac{N-1}{\sum_{j \neq i} d_{ij}}.$$

2 Results

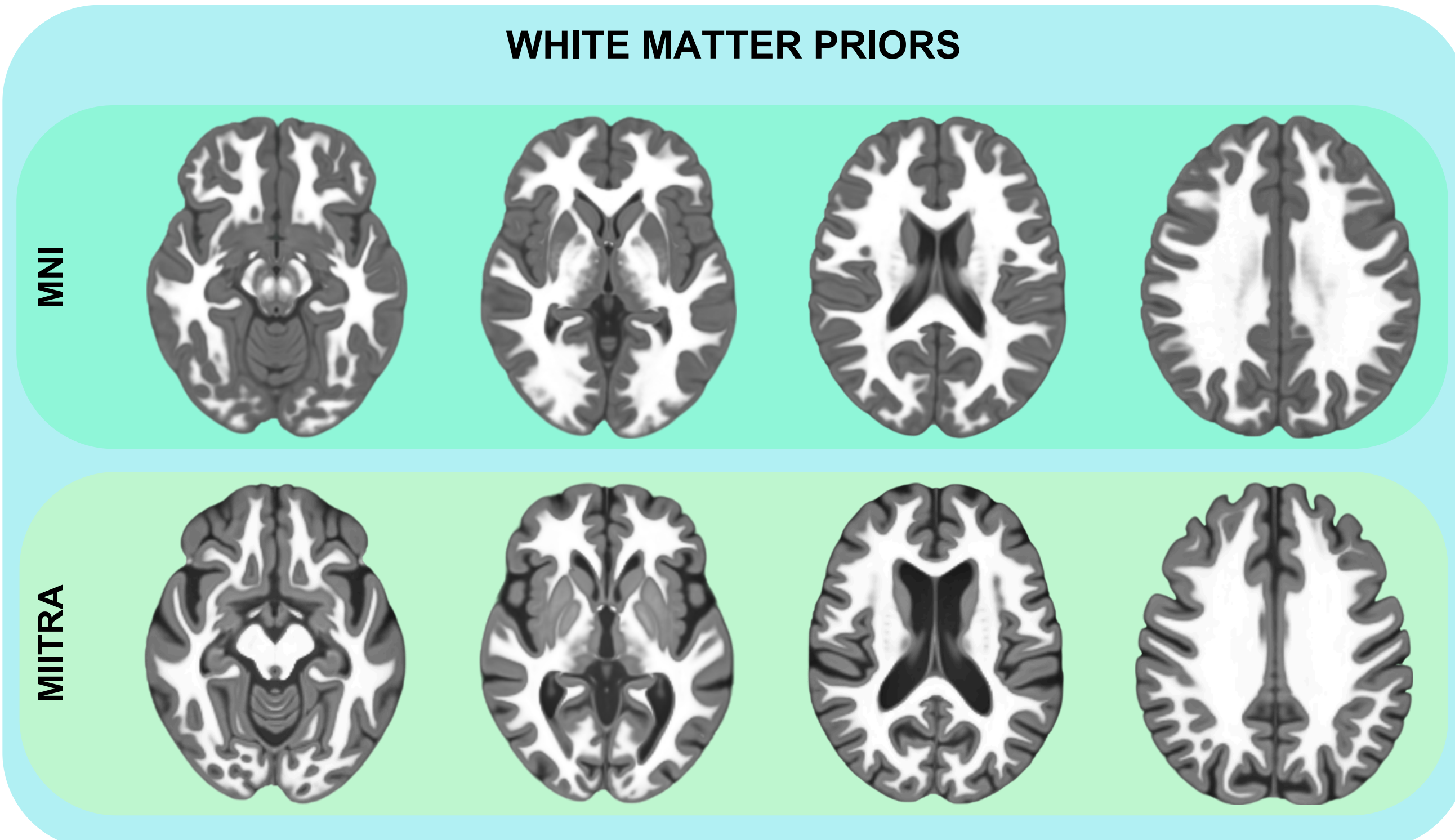

Figure S1: Comparison of WM priors between MNI template (top) and MIITRA template (bottom).

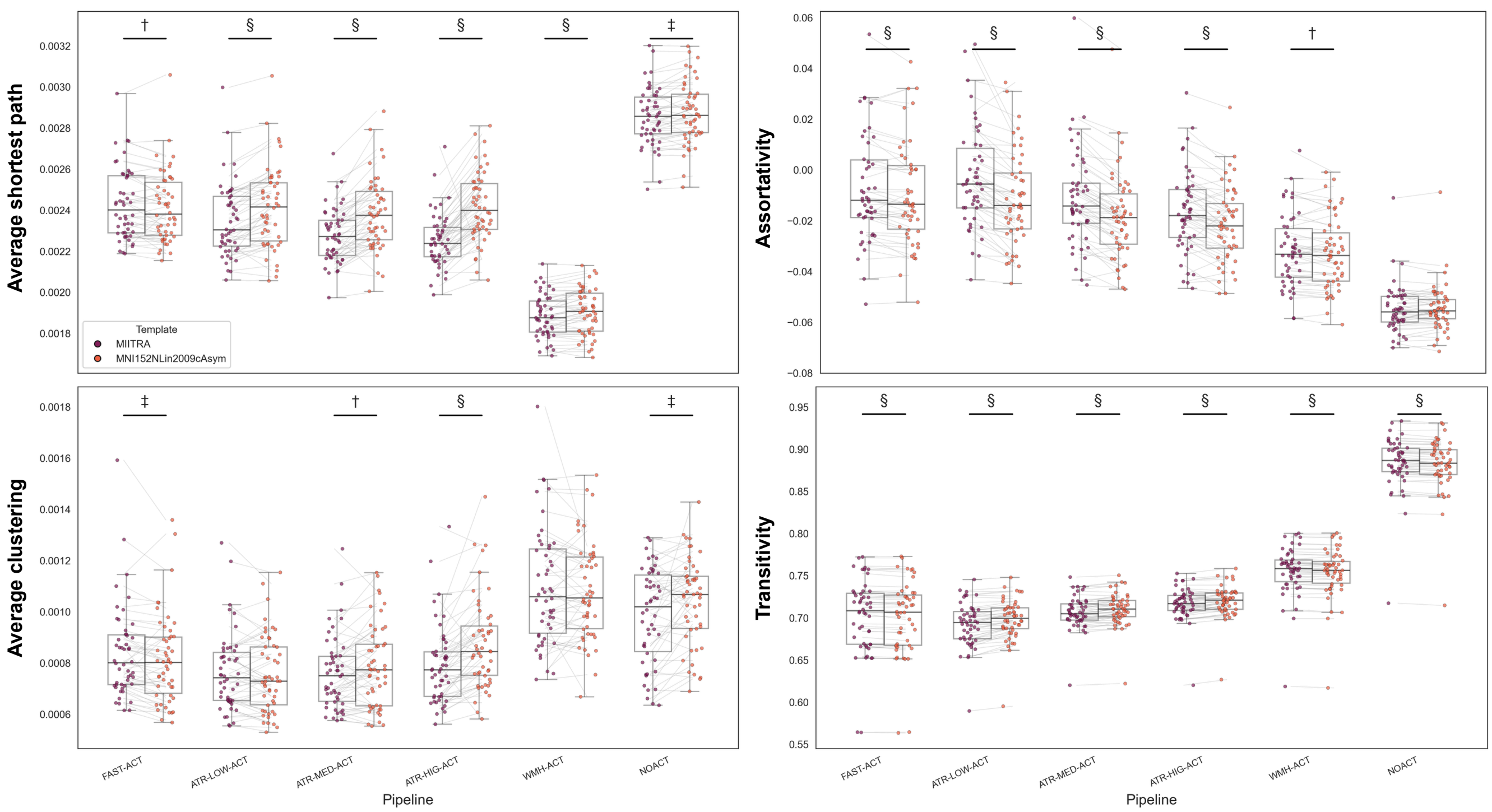

Figure S2: Comparison of global connectome metrics across pipelines and registration templates. Top-left: average shortest path (ASP), Top-right: assortativity (AS), Bottom-left: average clustering (ACL), Bottom-right: transitivity (TR). Symbols denote significance levels from paired t-tests between templates within each pipeline ($^{\dagger}q < 0.05$, $^{\ddagger}q < 0.01$, $^{\S}q < 0.001$).)

Table S1: Mean template effect per metric and pipeline with 95% CI and FDR-corrected q-values. Significance levels: † $q < 0.05$, ‡ $q < 0.01$, § $q < 0.001$. SP: sparsity, MC: mean connectivity, AST: assigned streamlines, GE: global efficiency, ASP: average shortest path, ACL: average clustering, AS: assortativity, TR: transitivity.

Pipeline	Metric	Δ (MIITRA–MNI)	95% CI	q-value
FAST-ACT	SP	$-1.73 \cdot 10^{-1}$	$[-2.38 \cdot 10^{-1}, -1.09 \cdot 10^{-1}]$	$3.37 \cdot 10^{-6}$§
	MC	$-4.40 \cdot 10^{-1}$	$[-6.34 \cdot 10^{-1}, -2.46 \cdot 10^{-1}]$	$4.89 \cdot 10^{-5}$§
	AST	$-3.06 \cdot 10^{-1}$	$[-3.70 \cdot 10^{-1}, -2.42 \cdot 10^{-1}]$	$3.23 \cdot 10^{-12}$§
	GE	-2.16	$[-4.63, 3.11 \cdot 10^{-1}]$	$4.26 \cdot 10^{-2}$†
	ASP	$1.67 \cdot 10^{-5}$	$[5.57 \cdot 10^{-7}, 3.28 \cdot 10^{-5}]$	$4.57 \cdot 10^{-2}$†
	ACL	$2.57 \cdot 10^{-5}$	$[5.41 \cdot 10^{-6}, 4.60 \cdot 10^{-5}]$	$9.68 \cdot 10^{-4}$§
	AS	$2.21 \cdot 10^{-3}$	$[1.06 \cdot 10^{-3}, 3.36 \cdot 10^{-3}]$	$4.45 \cdot 10^{-4}$§
	TR	$1.39 \cdot 10^{-3}$	$[9.11 \cdot 10^{-4}, 1.86 \cdot 10^{-3}]$	$7.00 \cdot 10^{-7}$§
ATR-LOW-ACT	SP	1.30	$[1.04, 1.56]$	$1.81 \cdot 10^{-13}$§
	MC	4.20	$[3.43, 4.97]$	$3.65 \cdot 10^{-14}$§
	AST	$3.65 \cdot 10^{-1}$	$[2.12 \cdot 10^{-1}, 5.19 \cdot 10^{-1}]$	$2.47 \cdot 10^{-5}$§
	GE	$1.53 \cdot 10^{1}$	$[1.16 \cdot 10^{1}, 1.90 \cdot 10^{1}]$	$2.28 \cdot 10^{-10}$§
	ASP	$-6.33 \cdot 10^{-5}$	$[-8.29 \cdot 10^{-5}, -4.36 \cdot 10^{-5}]$	$9.51 \cdot 10^{-8}$§
	ACL	$8.37 \cdot 10^{-6}$	$[-1.09 \cdot 10^{-5}, 2.77 \cdot 10^{-5}]$	$3.96 \cdot 10^{-1}$
	AS	$8.63 \cdot 10^{-3}$	$[6.23 \cdot 10^{-3}, 1.10 \cdot 10^{-2}]$	$7.91 \cdot 10^{-11}$§
	TR	$-6.29 \cdot 10^{-3}$	$[-7.74 \cdot 10^{-3}, -4.84 \cdot 10^{-3}]$	$3.81 \cdot 10^{-12}$§
ATR-MED-ACT	SP	$6.96 \cdot 10^{-1}$	$[5.03 \cdot 10^{-1}, 8.89 \cdot 10^{-1}]$	$6.54 \cdot 10^{-9}$§
	MC	3.53	$[2.91, 4.15]$	$1.54 \cdot 10^{-14}$§
	AST	$9.73 \cdot 10^{-1}$	$[7.86 \cdot 10^{-1}, 1.16]$	$2.18 \cdot 10^{-13}$§
	GE	$2.40 \cdot 10^{1}$	$[1.94 \cdot 10^{1}, 2.85 \cdot 10^{1}]$	$1.81 \cdot 10^{-13}$§
	ASP	$-1.04 \cdot 10^{-4}$	$[-1.30 \cdot 10^{-4}, -7.88 \cdot 10^{-5}]$	$2.28 \cdot 10^{-10}$§
	ACL	$-2.64 \cdot 10^{-5}$	$[-5.06 \cdot 10^{-5}, -2.23 \cdot 10^{-6}]$	$3.68 \cdot 10^{-2}$†
	AS	$7.23 \cdot 10^{-3}$	$[5.62 \cdot 10^{-3}, 8.84 \cdot 10^{-3}]$	$1.92 \cdot 10^{-11}$§
	TR	$-3.74 \cdot 10^{-3}$	$[-4.80 \cdot 10^{-3}, -2.68 \cdot 10^{-3}]$	$9.86 \cdot 10^{-9}$§
ATR-HIG-ACT	SP	$2.71 \cdot 10^{-1}$	$[8.02 \cdot 10^{-2}, 4.62 \cdot 10^{-1}]$	$7.58 \cdot 10^{-3}$‡
	MC	3.46	$[2.90, 4.02]$	$1.23 \cdot 10^{-15}$§
	AST	1.68	$[1.47, 1.89]$	$1.37 \cdot 10^{-19}$§
	GE	$3.71 \cdot 10^{1}$	$[3.26 \cdot 10^{1}, 4.17 \cdot 10^{1}]$	$7.26 \cdot 10^{-20}$§
	ASP	$-1.64 \cdot 10^{-4}$	$[-2.02 \cdot 10^{-4}, -1.26 \cdot 10^{-4}]$	$2.28 \cdot 10^{-10}$§
	ACL	$-8.42 \cdot 10^{-5}$	$[-1.16 \cdot 10^{-4}, -5.21 \cdot 10^{-5}]$	$4.13 \cdot 10^{-7}$§
	AS	$4.77 \cdot 10^{-3}$	$[3.02 \cdot 10^{-3}, 6.53 \cdot 10^{-3}]$	$2.65 \cdot 10^{-6}$§
	TR	$-3.13 \cdot 10^{-3}$	$[-4.39 \cdot 10^{-3}, -1.87 \cdot 10^{-3}]$	$1.22 \cdot 10^{-5}$§
WMH-ACT	SP	$-1.86 \cdot 10^{-1}$	$[-2.55 \cdot 10^{-1}, -1.17 \cdot 10^{-1}]$	$3.37 \cdot 10^{-6}$§
	MC	$-2.51 \cdot 10^{-1}$	$[-4.26 \cdot 10^{-1}, -7.72 \cdot 10^{-2}]$	$6.85 \cdot 10^{-3}$‡
	AST	$-2.15 \cdot 10^{-1}$	$[-2.51 \cdot 10^{-1}, -1.78 \cdot 10^{-1}]$	$6.64 \cdot 10^{-15}$§
	GE	5.74	$[3.57, 7.91]$	$4.12 \cdot 10^{-6}$§
	ASP	$-2.01 \cdot 10^{-5}$	$[-2.93 \cdot 10^{-5}, -1.09 \cdot 10^{-5}]$	$1.84 \cdot 10^{-4}$§
	ACL	$1.26 \cdot 10^{-5}$	$[-2.25 \cdot 10^{-5}, 4.77 \cdot 10^{-5}]$	$4.90 \cdot 10^{-1}$
	AS	$1.01 \cdot 10^{-3}$	$[1.29 \cdot 10^{-4}, 1.89 \cdot 10^{-3}]$	$2.90 \cdot 10^{-2}$†
	TR	$1.48 \cdot 10^{-3}$	$[1.09 \cdot 10^{-3}, 1.87 \cdot 10^{-3}]$	$1.61 \cdot 10^{-9}$§
NOACT	SP	$-2.20 \cdot 10^{-1}$	$[-2.93 \cdot 10^{-1}, -1.48 \cdot 10^{-1}]$	$3.17 \cdot 10^{-7}$§
	MC	$-3.53 \cdot 10^{-1}$	$[-4.40 \cdot 10^{-1}, -2.66 \cdot 10^{-1}]$	$3.39 \cdot 10^{-10}$§
	AST	$-3.14 \cdot 10^{-1}$	$[-3.60 \cdot 10^{-1}, -2.69 \cdot 10^{-1}]$	$3.65 \cdot 10^{-14}$§
	GE	2.80	$[1.12, 4.47]$	$1.94 \cdot 10^{-3}$‡
	ASP	$-1.86 \cdot 10^{-5}$	$[-3.20 \cdot 10^{-5}, -5.15 \cdot 10^{-6}]$	$8.96 \cdot 10^{-3}$‡
	ACL	$-5.82 \cdot 10^{-5}$	$[-9.21 \cdot 10^{-5}, -2.44 \cdot 10^{-5}]$	$1.73 \cdot 10^{-3}$‡
	AS	$-4.48 \cdot 10^{-4}$	$[-1.08 \cdot 10^{-3}, 1.84 \cdot 10^{-4}]$	$1.68 \cdot 10^{-1}$
	TR	$1.80 \cdot 10^{-3}$	$[1.31 \cdot 10^{-3}, 2.29 \cdot 10^{-3}]$	$3.59 \cdot 10^{-9}$§

Metric	Measure			
	MMSE	MoCA	Aβ-Cent	WMHB
SP	≈0§	$7.49 \cdot 10^{-2}$	≈0§	≈0§
MC	≈0§	$8.34 \cdot 10^{-1}$	≈0§	≈0§
AST	≈0§	$5.28 \cdot 10^{-3}$‡	≈0§	≈0§
GE	$2.94 \cdot 10^{-12}$§	$3.50 \cdot 10^{-3}$‡	$2.72 \cdot 10^{-3}$‡	$2.58 \cdot 10^{-14}$§
ASP	$1.55 \cdot 10^{-10}$§	$2.72 \cdot 10^{-3}$‡	$1.34 \cdot 10^{-3}$‡	≈0§
ACL	$2.72 \cdot 10^{-3}$‡	$2.49 \cdot 10^{-1}$	$2.49 \cdot 10^{-1}$	$3.66 \cdot 10^{-10}$§
AS	$6.83 \cdot 10^{-10}$§	$8.56 \cdot 10^{-2}$	$9.89 \cdot 10^{-6}$§	≈0§
TR	≈0§	$1.05 \cdot 10^{-1}$	$3.93 \cdot 10^{-11}$§	≈0§

Table S2: Omnibus tests (measure × pipeline) for global network metrics. Values are FDR-corrected q-values with significance levels denoted (†$q < 0.05$, ‡$q < 0.01$, §$q < 0.001$). SP: sparsity, MC: mean connectivity, AST: assigned streamlines, GE: global efficiency, ASP: average shortest path, ACL: average clustering, AS: assortativity, TR: transitivity.

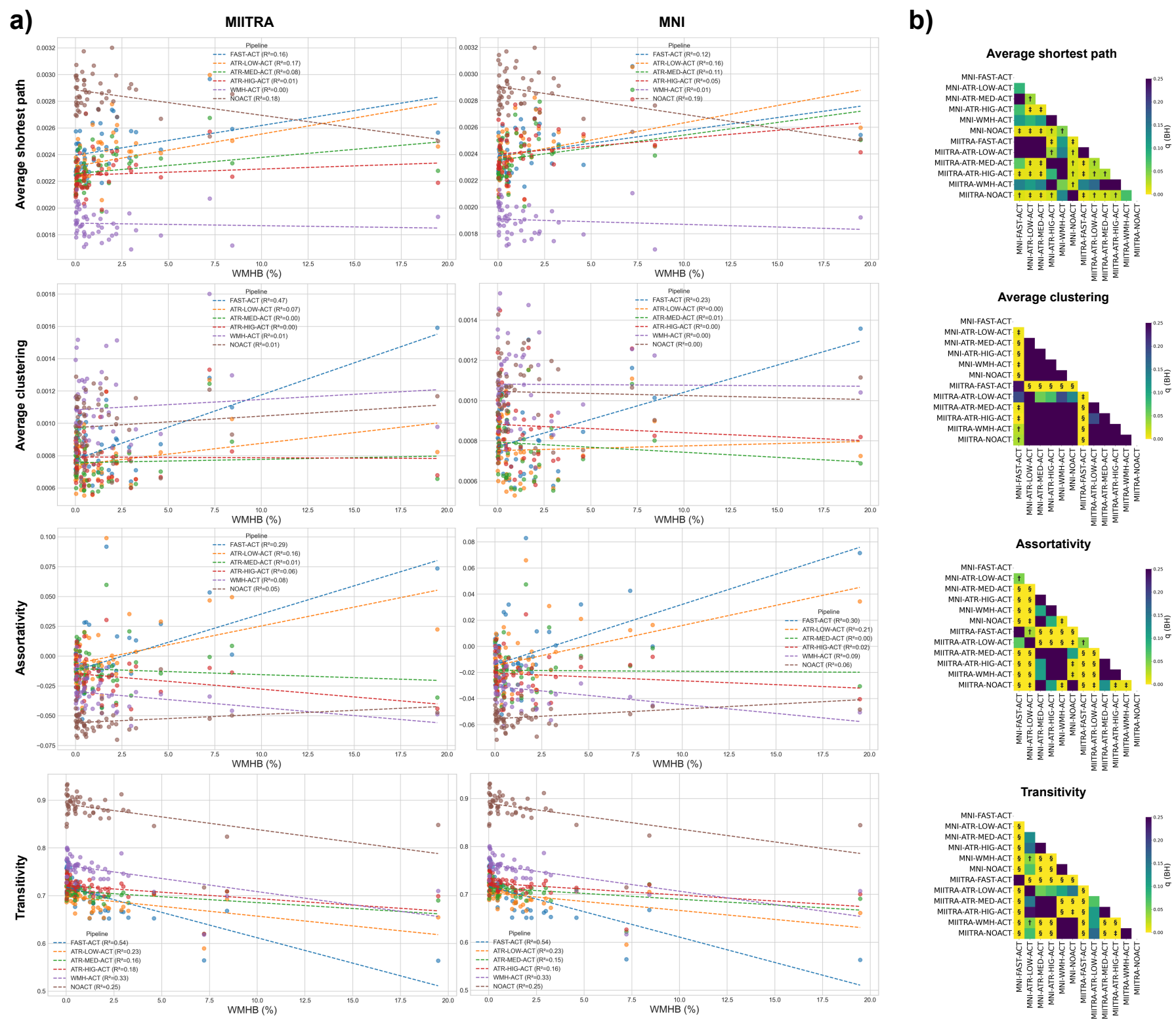

Figure S3: **a)** Scatter plots for four global metrics (in top to bottom order: average shortest path (ASP), average clustering (ACL), assortativity (AS), Transitivity (TR) shown separately by template (left: MIITRA; right: MNI) where each point is a subject (x-axis: white matter hyperintensity burden (WMHB); y-axis: metric). Dashed lines indicate per-pipeline linear fits and legends report R^2 for each fit. **b)** Lower-triangular heatmaps of FDR-corrected q-values (Benjamini–Hochberg) for pairwise differences between the slopes estimated in (a). Cells are annotated with significance markers ($^{\dagger}q < 0.05$, $^{\ddagger}q < 0.01$, $^{\S}q < 0.001$).

Table S3: Linear associations between global metrics and WMHB (Number of subjects = 50) by template and pipeline (slope with 95% CI and R^2). SP: sparsity, MC: mean connectivity, AST: assigned streamlines, GE: global efficiency, ASP: average shortest path, ACL: average clustering, AS: assortativity, TR: transitivity.

Clinical	Pipeline	Metric	MIITRA			MNI		
			Slope	95% CI	R^2	Slope	95% CI	R^2
WMHB	`FAST-ACT`	SP	2.40	$[1.88,2.91]$	0.689	2.40	$[1.89,2.92]$	0.690
		MC	6.25	$[5.42,7.08]$	0.874	6.36	$[5.53,7.19]$	0.878
		AST	$-6.69 \cdot 10^{-1}$	$[-8.85 \cdot 10^{-1},-4.54 \cdot 10^{-1}]$	0.390	$-6.58 \cdot 10^{-1}$	$[-8.74 \cdot 10^{-1},-4.43 \cdot 10^{-1}]$	0.381
		GE	-3.48	$[-6.39,-5.62 \cdot 10^{-1}]$	0.116	-3.05	$[-5.97,-1.37 \cdot 10^{-1}]$	0.090
		ASP	$1.94 \cdot 10^{-5}$	$[-5.17 \cdot 10^{-6},4.40 \cdot 10^{-5}]$	0.161	$1.63 \cdot 10^{-5}$	$[-6.49 \cdot 10^{-6},3.91 \cdot 10^{-5}]$	0.119
		ACL	$4.66 \cdot 10^{-5}$	$[2.99 \cdot 10^{-5},6.33 \cdot 10^{-5}]$	0.468	$3.38 \cdot 10^{-5}$	$[1.71 \cdot 10^{-5},5.05 \cdot 10^{-5}]$	0.232
		AS	$5.31 \cdot 10^{-3}$	$[3.61 \cdot 10^{-3},7.00 \cdot 10^{-3}]$	0.293	$5.20 \cdot 10^{-3}$	$[3.50 \cdot 10^{-3},6.89 \cdot 10^{-3}]$	0.296
		TR	$-9.70 \cdot 10^{-3}$	$[-1.22 \cdot 10^{-2},-7.16 \cdot 10^{-3}]$	0.543	$-9.66 \cdot 10^{-3}$	$[-1.22 \cdot 10^{-2},-7.12 \cdot 10^{-3}]$	0.542
	`ATR-LOW-ACT`	SP	1.28	$[7.65 \cdot 10^{-1},1.80]$	0.453	1.27	$[7.54 \cdot 10^{-1},1.78]$	0.491
		MC	2.59	$[1.76,3.43]$	0.459	2.30	$[1.47,3.13]$	0.434
		AST	$-4.50 \cdot 10^{-1}$	$[-6.66 \cdot 10^{-1},-2.35 \cdot 10^{-1}]$	0.276	$-4.44 \cdot 10^{-1}$	$[-6.60 \cdot 10^{-1},-2.28 \cdot 10^{-1}]$	0.273
		GE	-3.88	$[-6.80,-9.68 \cdot 10^{-1}]$	0.122	-4.32	$[-7.24,-1.41]$	0.126
		ASP	$2.11 \cdot 10^{-5}$	$[-8.17 \cdot 10^{-6},5.04 \cdot 10^{-5}]$	0.171	$2.30 \cdot 10^{-5}$	$[-3.68 \cdot 10^{-6},4.96 \cdot 10^{-5}]$	0.159
		ACL	$2.01 \cdot 10^{-5}$	$[3.41 \cdot 10^{-6},3.68 \cdot 10^{-5}]$	0.073	$9.13 \cdot 10^{-6}$	$[-7.56 \cdot 10^{-6},2.58 \cdot 10^{-5}]$	0.002
		AS	$3.74 \cdot 10^{-3}$	$[2.05 \cdot 10^{-3},5.44 \cdot 10^{-3}]$	0.164	$3.66 \cdot 10^{-3}$	$[1.96 \cdot 10^{-3},5.35 \cdot 10^{-3}]$	0.206
		TR	$-3.17 \cdot 10^{-3}$	$[-5.70 \cdot 10^{-3},-6.30 \cdot 10^{-4}]$	0.229	$-2.82 \cdot 10^{-3}$	$[-5.36 \cdot 10^{-3},-2.81 \cdot 10^{-4}]$	0.227
	`ATR-MED-ACT`	SP	$6.78 \cdot 10^{-1}$	$[1.63 \cdot 10^{-1},1.19]$	0.271	$7.42 \cdot 10^{-1}$	$[2.27 \cdot 10^{-1},1.26]$	0.304
		MC	1.47	$[6.38 \cdot 10^{-1},2.30]$	0.278	1.31	$[4.73 \cdot 10^{-1},2.14]$	0.232
		AST	$-1.04 \cdot 10^{-1}$	$[-3.19 \cdot 10^{-1},1.12 \cdot 10^{-1}]$	0.108	$-1.77 \cdot 10^{-1}$	$[-3.93 \cdot 10^{-1},3.87 \cdot 10^{-2}]$	0.154
		GE	-1.78	$[-4.69,1.14]$	0.039	-3.12	$[-6.04,-2.03 \cdot 10^{-1}]$	0.086
		ASP	$9.15 \cdot 10^{-6}$	$[-1.11 \cdot 10^{-5},2.94 \cdot 10^{-5}]$	0.081	$1.60 \cdot 10^{-5}$	$[-5.25 \cdot 10^{-6},3.72 \cdot 10^{-5}]$	0.113
		ACL	$8.91 \cdot 10^{-6}$	$[-7.78 \cdot 10^{-6},2.56 \cdot 10^{-5}]$	0.002	$1.79 \cdot 10^{-6}$	$[-1.49 \cdot 10^{-5},1.85 \cdot 10^{-5}]$	0.009
		AS	$7.01 \cdot 10^{-5}$	$[-1.62 \cdot 10^{-3},1.76 \cdot 10^{-3}]$	0.007	$5.00 \cdot 10^{-4}$	$[-1.19 \cdot 10^{-3},2.19 \cdot 10^{-3}]$	0.000
		TR	$-1.55 \cdot 10^{-3}$	$[-4.09 \cdot 10^{-3},9.85 \cdot 10^{-4}]$	0.157	$-1.47 \cdot 10^{-3}$	$[-4.01 \cdot 10^{-3},1.06 \cdot 10^{-3}]$	0.145
	`ATR-HIG-ACT`	SP	$5.90 \cdot 10^{-1}$	$[7.53 \cdot 10^{-2},1.11]$	0.245	$6.40 \cdot 10^{-1}$	$[1.25 \cdot 10^{-1},1.15]$	0.269
		MC	1.39	$[5.55 \cdot 10^{-1},2.22]$	0.273	1.27	$[4.39 \cdot 10^{-1},2.10]$	0.256
		AST	$3.02 \cdot 10^{-2}$	$[-1.86 \cdot 10^{-1},2.46 \cdot 10^{-1}]$	0.040	$-3.87 \cdot 10^{-2}$	$[-2.54 \cdot 10^{-1},1.77 \cdot 10^{-1}]$	0.076
		GE	$-5.63 \cdot 10^{-1}$	$[-3.48,2.35]$	0.005	-1.64	$[-4.56,1.28]$	0.028
		ASP	$1.83 \cdot 10^{-6}$	$[-1.38 \cdot 10^{-5},1.75 \cdot 10^{-5}]$	0.013	$9.02 \cdot 10^{-6}$	$[-1.17 \cdot 10^{-5},2.97 \cdot 10^{-5}]$	0.049
		ACL	$6.33 \cdot 10^{-6}$	$[-1.04 \cdot 10^{-5},2.30 \cdot 10^{-5}]$	0.000	$2.72 \cdot 10^{-6}$	$[-1.40 \cdot 10^{-5},1.94 \cdot 10^{-5}]$	0.005
		AS	$-7.29 \cdot 10^{-4}$	$[-2.42 \cdot 10^{-3},9.65 \cdot 10^{-4}]$	0.064	$-1.12 \cdot 10^{-5}$	$[-1.71 \cdot 10^{-3},1.68 \cdot 10^{-3}]$	0.015
		TR	$-1.77 \cdot 10^{-3}$	$[-4.31 \cdot 10^{-3},7.63 \cdot 10^{-4}]$	0.180	$-1.58 \cdot 10^{-3}$	$[-4.12 \cdot 10^{-3},9.57 \cdot 10^{-4}]$	0.165
	`WMH-ACT`	SP	$8.34 \cdot 10^{-1}$	$[3.19 \cdot 10^{-1},1.35]$	0.317	$8.50 \cdot 10^{-1}$	$[3.35 \cdot 10^{-1},1.36]$	0.320
		MC	1.38	$[5.48 \cdot 10^{-1},2.21]$	0.265	1.44	$[6.05 \cdot 10^{-1},2.27]$	0.273
		AST	$-1.56 \cdot 10^{-2}$	$[-2.31 \cdot 10^{-1},2.00 \cdot 10^{-1}]$	0.322	$-8.79 \cdot 10^{-3}$	$[-2.24 \cdot 10^{-1},2.07 \cdot 10^{-1}]$	0.314
		GE	$7.40 \cdot 10^{-1}$	$[-2.18,3.66]$	0.005	1.17	$[-1.75,4.08]$	0.012
		ASP	$-4.78 \cdot 10^{-6}$	$[-1.55 \cdot 10^{-5},5.94 \cdot 10^{-6}]$	0.003	$-6.89 \cdot 10^{-6}$	$[-1.86 \cdot 10^{-5},4.83 \cdot 10^{-6}]$	0.012
		ACL	$1.32 \cdot 10^{-5}$	$[-3.51 \cdot 10^{-6},2.99 \cdot 10^{-5}]$	0.008	$6.27 \cdot 10^{-6}$	$[-1.04 \cdot 10^{-5},2.30 \cdot 10^{-5}]$	0.000
		AS	$-7.58 \cdot 10^{-4}$	$[-2.45 \cdot 10^{-3},9.36 \cdot 10^{-4}]$	0.083	$-7.99 \cdot 10^{-4}$	$[-2.49 \cdot 10^{-3},8.95 \cdot 10^{-4}]$	0.092
		TR	$-4.57 \cdot 10^{-3}$	$[-7.11 \cdot 10^{-3},-2.03 \cdot 10^{-3}]$	0.326	$-4.62 \cdot 10^{-3}$	$[-7.16 \cdot 10^{-3},-2.08 \cdot 10^{-3}]$	0.327
	`NOACT`	SP	$6.27 \cdot 10^{-1}$	$[1.12 \cdot 10^{-1},1.14]$	0.265	$6.33 \cdot 10^{-1}$	$[1.18 \cdot 10^{-1},1.15]$	0.266
		MC	$8.84 \cdot 10^{-1}$	$[5.24 \cdot 10^{-2},1.72]$	0.286	$9.17 \cdot 10^{-1}$	$[8.54 \cdot 10^{-2},1.75]$	0.295
		AST	$3.54 \cdot 10^{-2}$	$[-1.80 \cdot 10^{-1},2.51 \cdot 10^{-1}]$	0.024	$5.23 \cdot 10^{-2}$	$[-1.63 \cdot 10^{-1},2.68 \cdot 10^{-1}]$	0.016
		GE	3.55	$[6.38 \cdot 10^{-1},6.47]$	0.285	3.73	$[8.11 \cdot 10^{-1},6.64]$	0.300
		ASP	$-2.19 \cdot 10^{-5}$	$[-3.27 \cdot 10^{-5},-1.10 \cdot 10^{-5}]$	0.175	$-2.39 \cdot 10^{-5}$	$[-3.35 \cdot 10^{-5},-1.43 \cdot 10^{-5}]$	0.193
		ACL	$1.39 \cdot 10^{-5}$	$[-2.77 \cdot 10^{-6},3.06 \cdot 10^{-5}]$	0.014	$4.77 \cdot 10^{-6}$	$[-1.19 \cdot 10^{-5},2.15 \cdot 10^{-5}]$	0.001
		AS	$1.27 \cdot 10^{-3}$	$[-4.29 \cdot 10^{-4},2.96 \cdot 10^{-3}]$	0.049	$1.32 \cdot 10^{-3}$	$[-3.72 \cdot 10^{-4},3.02 \cdot 10^{-3}]$	0.060
		TR	$-4.38 \cdot 10^{-3}$	$[-6.92 \cdot 10^{-3},-1.84 \cdot 10^{-3}]$	0.251	$-4.43 \cdot 10^{-3}$	$[-6.97 \cdot 10^{-3},-1.90 \cdot 10^{-3}]$	0.253

Table S4: Linear associations between global metrics and MMSE (Number of subjects = 50) by template and pipeline (slope with 95% CI and R^2). SP: sparsity, MC: mean connectivity, AST: assigned streamlines, GE: global efficiency, ASP: average shortest path, ACL: average clustering, AS: assortativity, TR: transitivity.

Clinical	Pipeline	Metric	MIITRA			MNI		
			Slope	95% CI	R^2	Slope	95% CI	R^2
MMSE	FAST-ACT	SP	-3.36	$[-4.73,-1.98]$	0.262	-3.36	$[-4.74,-1.99]$	0.262
		MC	-6.94	$[-9.56,-4.31]$	0.220	-7.15	$[-9.77,-4.52]$	0.225
		AST	1.55	$[1.06,2.04]$	0.323	1.51	$[1.01,2.00]$	0.310
		GE	8.93	$[3.31,1.46 \cdot 10^{1}]$	0.118	7.26	$[1.79,1.27 \cdot 10^{1}]$	0.078
		ASP	$-4.44 \cdot 10^{-5}$	$[-7.25 \cdot 10^{-5},-1.62 \cdot 10^{-5}]$	0.124	$-3.25 \cdot 10^{-5}$	$[-5.95 \cdot 10^{-5},-5.48 \cdot 10^{-6}]$	0.070
		ACL	$-2.50 \cdot 10^{-5}$	$[-6.53 \cdot 10^{-5},1.54 \cdot 10^{-5}]$	0.021	$1.95 \cdot 10^{-6}$	$[-3.84 \cdot 10^{-5},4.23 \cdot 10^{-5}]$	0.002
		AS	$-5.53 \cdot 10^{-3}$	$[-9.78 \cdot 10^{-3},-1.28 \cdot 10^{-3}]$	0.044	$-5.71 \cdot 10^{-3}$	$[-9.96 \cdot 10^{-3},-1.46 \cdot 10^{-3}]$	0.050
		TR	$1.42 \cdot 10^{-2}$	$[7.75 \cdot 10^{-3},2.07 \cdot 10^{-2}]$	0.222	$1.41 \cdot 10^{-2}$	$[7.65 \cdot 10^{-3},2.06 \cdot 10^{-2}]$	0.221
	ATR-LOW-ACT	SP	-1.60	$[-2.98,-2.31 \cdot 10^{-1}]$	0.167	-1.43	$[-2.81,-6.17 \cdot 10^{-2}]$	0.158
		MC	-1.72	$[-4.35,9.06 \cdot 10^{-1}]$	0.076	$-9.92 \cdot 10^{-1}$	$[-3.62,1.63]$	0.049
		AST	1.23	$[7.32 \cdot 10^{-1},1.72]$	0.293	1.20	$[7.10 \cdot 10^{-1},1.70]$	0.288
		GE	8.39	$[2.93,1.39 \cdot 10^{1}]$	0.088	$1.00 \cdot 10^{1}$	$[4.60,1.55 \cdot 10^{1}]$	0.105
		ASP	$-3.72 \cdot 10^{-5}$	$[-6.31 \cdot 10^{-5},-1.13 \cdot 10^{-5}]$	0.083	$-4.36 \cdot 10^{-5}$	$[-7.03 \cdot 10^{-5},-1.70 \cdot 10^{-5}]$	0.088
		ACL	$1.55 \cdot 10^{-5}$	$[-2.49 \cdot 10^{-5},5.59 \cdot 10^{-5}]$	0.025	$3.97 \cdot 10^{-5}$	$[-6.20 \cdot 10^{-7},8.01 \cdot 10^{-5}]$	0.130
		AS	$-4.74 \cdot 10^{-3}$	$[-8.99 \cdot 10^{-3},-4.89 \cdot 10^{-4}]$	0.038	$-4.56 \cdot 10^{-3}$	$[-8.81 \cdot 10^{-3},-3.07 \cdot 10^{-4}]$	0.045
		TR	$3.15 \cdot 10^{-3}$	$[-3.33 \cdot 10^{-3},9.63 \cdot 10^{-3}]$	0.075	$1.99 \cdot 10^{-3}$	$[-4.49 \cdot 10^{-3},8.47 \cdot 10^{-3}]$	0.057
	ATR-MED-ACT	SP	$-2.70 \cdot 10^{-1}$	$[-1.64,1.10]$	0.048	$-3.76 \cdot 10^{-1}$	$[-1.75,9.97 \cdot 10^{-1}]$	0.058
		MC	$1.73 \cdot 10^{-1}$	$[-2.45,2.80]$	0.013	$4.44 \cdot 10^{-1}$	$[-2.18,3.07]$	0.007
		AST	$4.89 \cdot 10^{-1}$	$[-4.75 \cdot 10^{-3},9.83 \cdot 10^{-1}]$	0.181	$6.36 \cdot 10^{-1}$	$[1.42 \cdot 10^{-1},1.13]$	0.214
		GE	4.27	$[-1.46,1.00 \cdot 10^{1}]$	0.033	6.09	$[6.36 \cdot 10^{-1},1.15 \cdot 10^{1}]$	0.050
		ASP	$-1.73 \cdot 10^{-5}$	$[-4.27 \cdot 10^{-5},8.17 \cdot 10^{-6}]$	0.042	$-2.32 \cdot 10^{-5}$	$[-5.07 \cdot 10^{-5},4.25 \cdot 10^{-6}]$	0.039
		ACL	$3.44 \cdot 10^{-5}$	$[-5.92 \cdot 10^{-6},7.48 \cdot 10^{-5}]$	0.110	$5.36 \cdot 10^{-5}$	$[1.33 \cdot 10^{-5},9.40 \cdot 10^{-5}]$	0.198
		AS	$1.28 \cdot 10^{-3}$	$[-2.97 \cdot 10^{-3},5.53 \cdot 10^{-3}]$	0.022	$9.26 \cdot 10^{-4}$	$[-3.32 \cdot 10^{-3},5.18 \cdot 10^{-3}]$	0.019
		TR	$-1.23 \cdot 10^{-3}$	$[-7.71 \cdot 10^{-3},5.25 \cdot 10^{-3}]$	0.009	$-1.17 \cdot 10^{-3}$	$[-7.65 \cdot 10^{-3},5.32 \cdot 10^{-3}]$	0.009
	ATR-HIG-ACT	SP	$-4.03 \cdot 10^{-2}$	$[-1.41,1.33]$	0.031	$-1.04 \cdot 10^{-1}$	$[-1.48,1.27]$	0.035
		MC	$2.52 \cdot 10^{-1}$	$[-2.37,2.88]$	0.012	$5.25 \cdot 10^{-1}$	$[-2.10,3.15]$	0.006
		AST	$1.82 \cdot 10^{-1}$	$[-3.11 \cdot 10^{-1},6.76 \cdot 10^{-1}]$	0.131	$3.41 \cdot 10^{-1}$	$[-1.53 \cdot 10^{-1},8.35 \cdot 10^{-1}]$	0.158
		GE	1.38	$[-4.38,7.13]$	0.004	3.22	$[-2.97,9.41]$	0.016
		ASP	$-3.10 \cdot 10^{-6}$	$[-2.84 \cdot 10^{-5},2.22 \cdot 10^{-5}]$	0.005	$-9.93 \cdot 10^{-6}$	$[-4.20 \cdot 10^{-5},2.21 \cdot 10^{-5}]$	0.011
		ACL	$3.85 \cdot 10^{-5}$	$[-1.82 \cdot 10^{-6},7.89 \cdot 10^{-5}]$	0.119	$4.39 \cdot 10^{-5}$	$[3.55 \cdot 10^{-6},8.43 \cdot 10^{-5}]$	0.107
		AS	$3.24 \cdot 10^{-3}$	$[-1.01 \cdot 10^{-3},7.49 \cdot 10^{-3}]$	0.106	$2.10 \cdot 10^{-3}$	$[-2.15 \cdot 10^{-3},6.35 \cdot 10^{-3}]$	0.066
		TR	$-1.19 \cdot 10^{-3}$	$[-7.67 \cdot 10^{-3},5.30 \cdot 10^{-3}]$	0.009	$-1.48 \cdot 10^{-3}$	$[-7.97 \cdot 10^{-3},5.00 \cdot 10^{-3}]$	0.006
	WMH-ACT	SP	$-5.33 \cdot 10^{-1}$	$[-1.91,8.40 \cdot 10^{-1}]$	0.066	$-5.48 \cdot 10^{-1}$	$[-1.92,8.25 \cdot 10^{-1}]$	0.067
		MC	$-2.69 \cdot 10^{-1}$	$[-2.89,2.36]$	0.032	$-3.73 \cdot 10^{-1}$	$[-3.00,2.25]$	0.036
		AST	$1.69 \cdot 10^{-1}$	$[-3.25 \cdot 10^{-1},6.62 \cdot 10^{-1}]$	0.458	$1.41 \cdot 10^{-1}$	$[-3.52 \cdot 10^{-1},6.35 \cdot 10^{-1}]$	0.432
		GE	-5.88	$[-1.41 \cdot 10^{1},2.31]$	0.061	-5.58	$[-1.36 \cdot 10^{1},2.41]$	0.049
		ASP	$2.60 \cdot 10^{-5}$	$[-4.27 \cdot 10^{-6},5.62 \cdot 10^{-5}]$	0.068	$2.59 \cdot 10^{-5}$	$[-4.46 \cdot 10^{-6},5.63 \cdot 10^{-5}]$	0.058
		ACL	$1.16 \cdot 10^{-5}$	$[-2.88 \cdot 10^{-5},5.19 \cdot 10^{-5}]$	0.007	$1.39 \cdot 10^{-5}$	$[-2.64 \cdot 10^{-5},5.43 \cdot 10^{-5}]$	0.014
		AS	$2.47 \cdot 10^{-3}$	$[-1.78 \cdot 10^{-3},6.72 \cdot 10^{-3}]$	0.089	$2.08 \cdot 10^{-3}$	$[-2.17 \cdot 10^{-3},6.33 \cdot 10^{-3}]$	0.073
		TR	$3.61 \cdot 10^{-3}$	$[-2.87 \cdot 10^{-3},1.01 \cdot 10^{-2}]$	0.069	$3.62 \cdot 10^{-3}$	$[-2.86 \cdot 10^{-3},1.01 \cdot 10^{-2}]$	0.069
	NOACT	SP	$-3.12 \cdot 10^{-1}$	$[-1.68,1.06]$	0.057	$-3.09 \cdot 10^{-1}$	$[-1.68,1.06]$	0.056
		MC	$1.86 \cdot 10^{-1}$	$[-2.44,2.81]$	0.033	$1.00 \cdot 10^{-1}$	$[-2.53,2.73]$	0.038
		AST	$2.57 \cdot 10^{-1}$	$[-2.37 \cdot 10^{-1},7.51 \cdot 10^{-1}]$	0.122	$1.84 \cdot 10^{-1}$	$[-3.10 \cdot 10^{-1},6.78 \cdot 10^{-1}]$	0.091
		GE	-3.27	$[-1.09 \cdot 10^{1},4.32]$	0.042	-4.02	$[-1.16 \cdot 10^{1},3.58]$	0.060
		ASP	$2.09 \cdot 10^{-5}$	$[-2.44 \cdot 10^{-5},6.63 \cdot 10^{-5}]$	0.022	$2.89 \cdot 10^{-5}$	$[-1.65 \cdot 10^{-5},7.44 \cdot 10^{-5}]$	0.042
		ACL	$5.68 \cdot 10^{-7}$	$[-3.98 \cdot 10^{-5},4.09 \cdot 10^{-5}]$	0.001	$3.86 \cdot 10^{-6}$	$[-3.65 \cdot 10^{-5},4.42 \cdot 10^{-5}]$	0.003
		AS	$-2.83 \cdot 10^{-3}$	$[-7.08 \cdot 10^{-3},1.42 \cdot 10^{-3}]$	0.058	$-3.04 \cdot 10^{-3}$	$[-7.29 \cdot 10^{-3},1.21 \cdot 10^{-3}]$	0.076
		TR	$3.07 \cdot 10^{-3}$	$[-3.42 \cdot 10^{-3},9.55 \cdot 10^{-3}]$	0.048	$3.03 \cdot 10^{-3}$	$[-3.46 \cdot 10^{-3},9.51 \cdot 10^{-3}]$	0.046

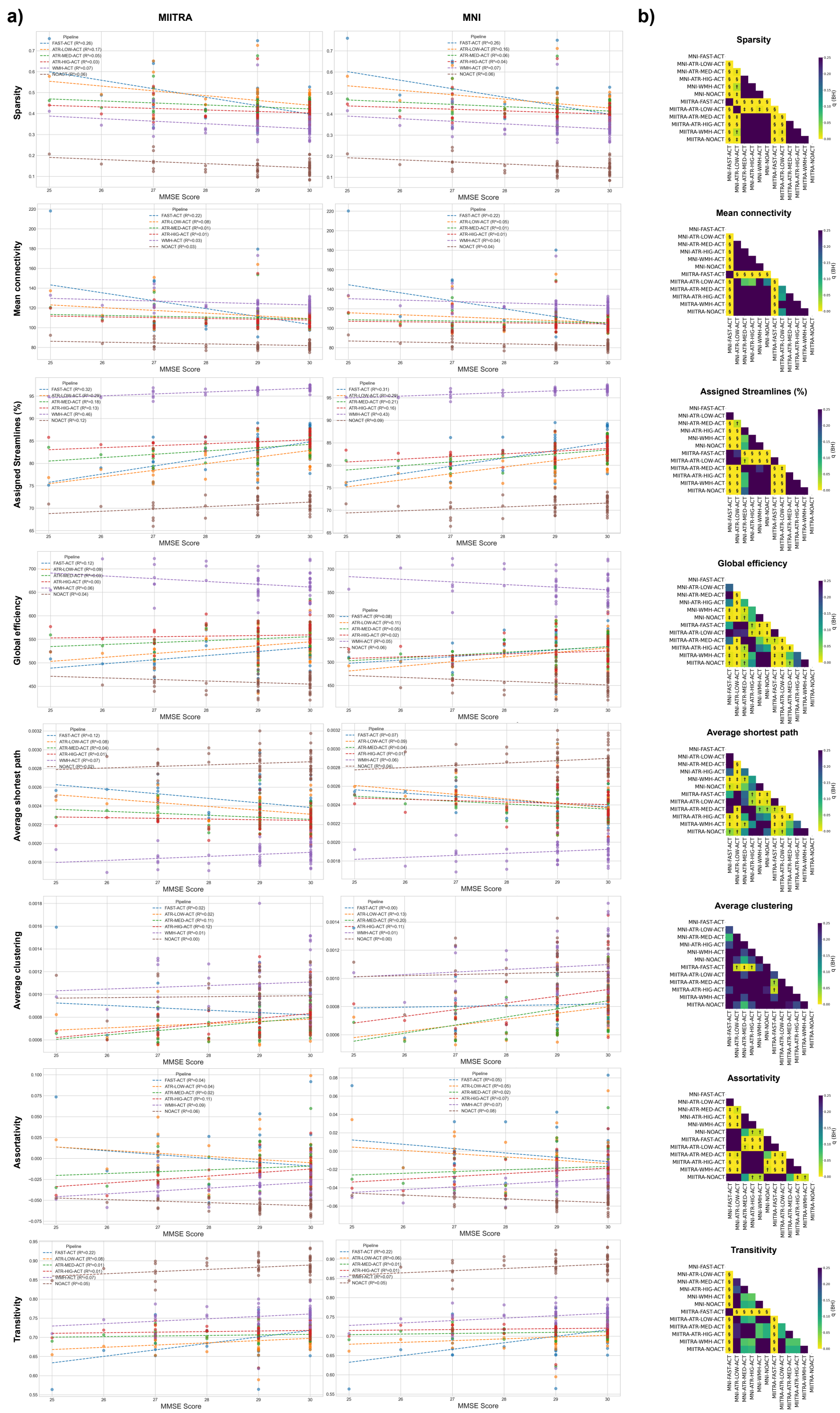

Figure S4: **a)** Scatter plots for seven global metrics (in top to bottom order: sparsity (SP), mean connectivity (MC), assigned streamlines (AST), global efficiency (GE), average shortest path (ASP), average clustering (ACL), assortativity (AS), transitivity (TR) shown separately by template (left: MIITRA; right: MNI) where each point is a subject (x-axis: MMSE Score; y-axis: metric). Dashed lines indicate per-pipeline linear fits and legends report R^2 for each fit. **b)** Lower-triangular heatmaps of FDR-corrected q-values (Benjamini–Hochberg) for pairwise differences between the slopes estimated in (a). Cells are annotated with significance markers ($^\dagger q < 0.05$, $^\ddagger q < 0.01$, $^\S q < 0.001$).

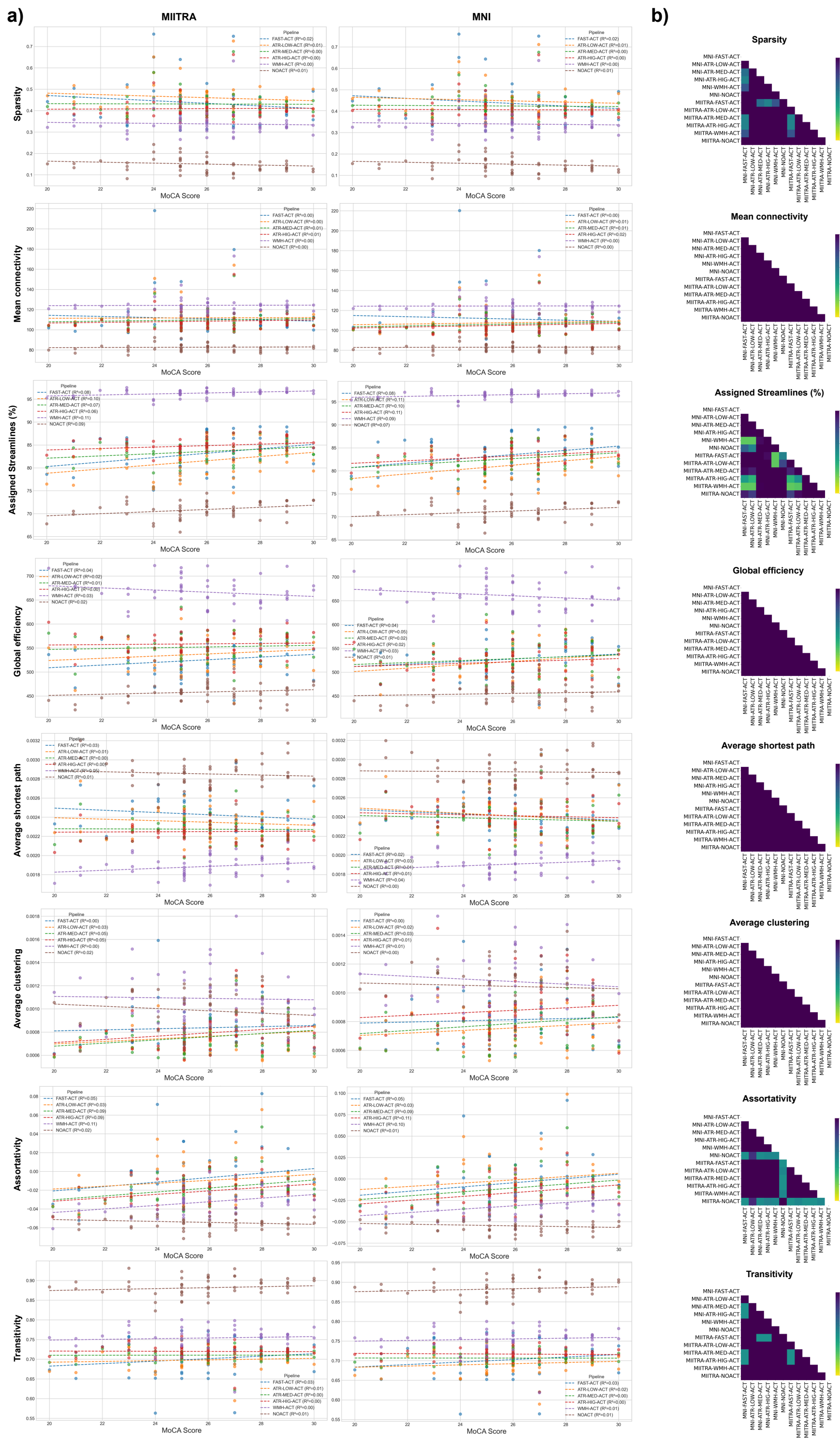

Figure S5: **a)** Scatter plots for seven global metrics (in top to bottom order: sparsity (SP), mean connectivity (MC), assigned streamlines (AST), global efficiency (GE), average shortest path (ASP), average clustering (ACL), assortativity (AS), transitivity (TR) shown separately by template (left: MIITRA; right: MNI) where each point is a subject (x-axis: MoCA Score; y-axis: metric). Dashed lines indicate per-pipeline linear fits and legends report R^2 for each fit. **b)** Lower-triangular heatmaps of FDR-corrected q-values (Benjamini–Hochberg) for pairwise differences between the slopes estimated in (a). Cells are annotated with significance markers ($^{\dagger}q < 0.05$, $^{\ddagger}q < 0.01$, $^{\S}q < 0.001$).

Table S5: Linear associations between global metrics and MoCA (Number of subjects = 49) by template and pipeline (slope with 95% CI and R^2). SP: sparsity, MC: mean connectivity, AST: assigned streamlines, GE: global efficiency, ASP: average shortest path, ACL: average clustering, AS: assortativity, TR: transitivity.

Clinical	Pipeline	Metric	MIITRA			MNI		
			Slope	95% CI	R^2	Slope	95% CI	R^2
MoCA	FAST-ACT	SP	$-7.29 \cdot 10^{-1}$	$[-1.51, 5.52 \cdot 10^{-2}]$	0.021	$-7.23 \cdot 10^{-1}$	$[-1.51, 6.12 \cdot 10^{-2}]$	0.021
		MC	$-7.91 \cdot 10^{-1}$	$[-2.29, 7.10 \cdot 10^{-1}]$	0.004	$-8.14 \cdot 10^{-1}$	$[-2.32, 6.87 \cdot 10^{-1}]$	0.004
		AST	$4.32 \cdot 10^{-1}$	$[1.44 \cdot 10^{-1}, 7.21 \cdot 10^{-1}]$	0.085	$4.13 \cdot 10^{-1}$	$[1.24 \cdot 10^{-1}, 7.02 \cdot 10^{-1}]$	0.079
		GE	2.93	$[-8.76 \cdot 10^{-1}, 6.74]$	0.043	2.76	$[-8.12 \cdot 10^{-1}, 6.34]$	0.038
		ASP	$-1.17 \cdot 10^{-5}$	$[-3.29 \cdot 10^{-5}, 9.55 \cdot 10^{-6}]$	0.026	$-1.07 \cdot 10^{-5}$	$[-3.01 \cdot 10^{-5}, 8.71 \cdot 10^{-6}]$	0.021
		ACL	$-9.50 \cdot 10^{-7}$	$[-2.33 \cdot 10^{-5}, 2.14 \cdot 10^{-5}]$	0.004	$-1.59 \cdot 10^{-6}$	$[-2.39 \cdot 10^{-5}, 2.07 \cdot 10^{-5}]$	0.003
		AS	$1.70 \cdot 10^{-3}$	$[-6.14 \cdot 10^{-4}, 4.02 \cdot 10^{-3}]$	0.046	$1.64 \cdot 10^{-3}$	$[-6.81 \cdot 10^{-4}, 3.95 \cdot 10^{-3}]$	0.046
		TR	$3.74 \cdot 10^{-3}$	$[9.25 \cdot 10^{-5}, 7.38 \cdot 10^{-3}]$	0.029	$3.69 \cdot 10^{-3}$	$[4.49 \cdot 10^{-5}, 7.33 \cdot 10^{-3}]$	0.028
	ATR-LOW-ACT	SP	$-4.74 \cdot 10^{-1}$	$[-1.26, 3.10 \cdot 10^{-1}]$	0.014	$-4.02 \cdot 10^{-1}$	$[-1.19, 3.82 \cdot 10^{-1}]$	0.010
		MC	$-1.44 \cdot 10^{-1}$	$[-1.65, 1.36]$	0.000	$1.31 \cdot 10^{-1}$	$[-1.37, 1.63]$	0.005
		AST	$4.03 \cdot 10^{-1}$	$[1.14 \cdot 10^{-1}, 6.91 \cdot 10^{-1}]$	0.100	$4.28 \cdot 10^{-1}$	$[1.40 \cdot 10^{-1}, 7.17 \cdot 10^{-1}]$	0.113
		GE	2.42	$[-1.64, 6.49]$	0.024	3.68	$[-3.42 \cdot 10^{-1}, 7.70]$	0.049
		ASP	$-7.93 \cdot 10^{-6}$	$[-3.03 \cdot 10^{-5}, 1.44 \cdot 10^{-5}]$	0.011	$-1.37 \cdot 10^{-5}$	$[-3.63 \cdot 10^{-5}, 8.96 \cdot 10^{-6}]$	0.026
		ACL	$5.47 \cdot 10^{-6}$	$[-1.69 \cdot 10^{-5}, 2.78 \cdot 10^{-5}]$	0.029	$3.19 \cdot 10^{-6}$	$[-1.91 \cdot 10^{-5}, 2.55 \cdot 10^{-5}]$	0.019
		AS	$1.14 \cdot 10^{-3}$	$[-1.17 \cdot 10^{-3}, 3.46 \cdot 10^{-3}]$	0.034	$8.08 \cdot 10^{-4}$	$[-1.51 \cdot 10^{-3}, 3.13 \cdot 10^{-3}]$	0.031
		TR	$2.05 \cdot 10^{-3}$	$[-1.60 \cdot 10^{-3}, 5.69 \cdot 10^{-3}]$	0.018	$1.51 \cdot 10^{-3}$	$[-2.13 \cdot 10^{-3}, 5.16 \cdot 10^{-3}]$	0.009
	ATR-MED-ACT	SP	$-1.50 \cdot 10^{-1}$	$[-9.35 \cdot 10^{-1}, 6.34 \cdot 10^{-1}]$	0.000	$-1.85 \cdot 10^{-1}$	$[-9.69 \cdot 10^{-1}, 5.99 \cdot 10^{-1}]$	0.001
		MC	$9.22 \cdot 10^{-2}$	$[-1.41, 1.59]$	0.006	$2.47 \cdot 10^{-1}$	$[-1.25, 1.75]$	0.014
		AST	$1.92 \cdot 10^{-1}$	$[-9.71 \cdot 10^{-2}, 4.80 \cdot 10^{-1}]$	0.070	$2.71 \cdot 10^{-1}$	$[-1.81 \cdot 10^{-2}, 5.59 \cdot 10^{-1}]$	0.102
		GE	1.00	$[-2.40, 4.40]$	0.005	2.18	$[-1.24, 5.61]$	0.021
		ASP	$-8.32 \cdot 10^{-7}$	$[-1.73 \cdot 10^{-5}, 1.57 \cdot 10^{-5}]$	0.000	$-5.94 \cdot 10^{-6}$	$[-2.43 \cdot 10^{-5}, 1.25 \cdot 10^{-5}]$	0.006
		ACL	$8.07 \cdot 10^{-6}$	$[-1.42 \cdot 10^{-5}, 3.04 \cdot 10^{-5}]$	0.051	$6.33 \cdot 10^{-6}$	$[-1.60 \cdot 10^{-5}, 2.87 \cdot 10^{-5}]$	0.031
		AS	$1.52 \cdot 10^{-3}$	$[-7.97 \cdot 10^{-4}, 3.84 \cdot 10^{-3}]$	0.086	$1.36 \cdot 10^{-3}$	$[-9.54 \cdot 10^{-4}, 3.68 \cdot 10^{-3}]$	0.091
		TR	$4.98 \cdot 10^{-4}$	$[-3.15 \cdot 10^{-3}, 4.14 \cdot 10^{-3}]$	0.000	$5.68 \cdot 10^{-4}$	$[-3.08 \cdot 10^{-3}, 4.21 \cdot 10^{-3}]$	0.000
	ATR-HIG-ACT	SP	$-7.54 \cdot 10^{-2}$	$[-8.60 \cdot 10^{-1}, 7.09 \cdot 10^{-1}]$	0.000	$-1.49 \cdot 10^{-1}$	$[-9.34 \cdot 10^{-1}, 6.35 \cdot 10^{-1}]$	0.000
		MC	$8.12 \cdot 10^{-2}$	$[-1.42, 1.58]$	0.006	$2.33 \cdot 10^{-1}$	$[-1.27, 1.73]$	0.015
		AST	$1.05 \cdot 10^{-1}$	$[-1.84 \cdot 10^{-1}, 3.94 \cdot 10^{-1}]$	0.061	$2.12 \cdot 10^{-1}$	$[-7.67 \cdot 10^{-2}, 5.01 \cdot 10^{-1}]$	0.114
		GE	$5.53 \cdot 10^{-1}$	$[-2.78, 3.88]$	0.001	1.84	$[-1.67, 5.34]$	0.017
		ASP	$1.04 \cdot 10^{-6}$	$[-1.41 \cdot 10^{-5}, 1.62 \cdot 10^{-5}]$	0.000	$-5.22 \cdot 10^{-6}$	$[-2.47 \cdot 10^{-5}, 1.43 \cdot 10^{-5}]$	0.005
		ACL	$8.52 \cdot 10^{-6}$	$[-1.38 \cdot 10^{-5}, 3.08 \cdot 10^{-5}]$	0.047	$2.97 \cdot 10^{-6}$	$[-1.93 \cdot 10^{-5}, 2.53 \cdot 10^{-5}]$	0.013
		AS	$1.47 \cdot 10^{-3}$	$[-8.51 \cdot 10^{-4}, 3.78 \cdot 10^{-3}]$	0.111	$1.08 \cdot 10^{-3}$	$[-1.23 \cdot 10^{-3}, 3.40 \cdot 10^{-3}]$	0.089
		TR	$2.24 \cdot 10^{-4}$	$[-3.42 \cdot 10^{-3}, 3.87 \cdot 10^{-3}]$	0.001	$3.71 \cdot 10^{-4}$	$[-3.27 \cdot 10^{-3}, 4.01 \cdot 10^{-3}]$	0.000
	WMH-ACT	SP	$-2.35 \cdot 10^{-1}$	$[-1.02, 5.49 \cdot 10^{-1}]$	0.002	$-2.25 \cdot 10^{-1}$	$[-1.01, 5.59 \cdot 10^{-1}]$	0.002
		MC	$-1.75 \cdot 10^{-1}$	$[-1.68, 1.33]$	0.000	$-1.86 \cdot 10^{-1}$	$[-1.69, 1.32]$	0.000
		AST	$5.66 \cdot 10^{-2}$	$[-2.32 \cdot 10^{-1}, 3.45 \cdot 10^{-1}]$	0.109	$4.28 \cdot 10^{-2}$	$[-2.46 \cdot 10^{-1}, 3.31 \cdot 10^{-1}]$	0.090
		GE	-2.09	$[-5.61, 1.44]$	0.031	-2.11	$[-5.85, 1.64]$	0.028
		ASP	$1.01 \cdot 10^{-5}$	$[-3.86 \cdot 10^{-6}, 2.42 \cdot 10^{-5}]$	0.055	$9.28 \cdot 10^{-6}$	$[-6.10 \cdot 10^{-6}, 2.47 \cdot 10^{-5}]$	0.040
		ACL	$-8.52 \cdot 10^{-6}$	$[-3.08 \cdot 10^{-5}, 1.38 \cdot 10^{-5}]$	0.001	$-1.46 \cdot 10^{-5}$	$[-3.69 \cdot 10^{-5}, 7.75 \cdot 10^{-6}]$	0.013
		AS	$1.18 \cdot 10^{-3}$	$[-1.13 \cdot 10^{-3}, 3.50 \cdot 10^{-3}]$	0.103	$1.17 \cdot 10^{-3}$	$[-1.15 \cdot 10^{-3}, 3.49 \cdot 10^{-3}]$	0.106
		TR	$1.49 \cdot 10^{-3}$	$[-2.16 \cdot 10^{-3}, 5.13 \cdot 10^{-3}]$	0.006	$1.42 \cdot 10^{-3}$	$[-2.22 \cdot 10^{-3}, 5.06 \cdot 10^{-3}]$	0.005
	NOACT	SP	$-3.49 \cdot 10^{-1}$	$[-1.13, 4.35 \cdot 10^{-1}]$	0.011	$-3.45 \cdot 10^{-1}$	$[-1.13, 4.39 \cdot 10^{-1}]$	0.010
		MC	$-1.55 \cdot 10^{-1}$	$[-1.66, 1.35]$	0.001	$-1.85 \cdot 10^{-1}$	$[-1.69, 1.32]$	0.000
		AST	$1.79 \cdot 10^{-1}$	$[-1.10 \cdot 10^{-1}, 4.67 \cdot 10^{-1}]$	0.089	$1.44 \cdot 10^{-1}$	$[-1.44 \cdot 10^{-1}, 4.33 \cdot 10^{-1}]$	0.066
		GE	1.34	$[-1.89, 4.57]$	0.019	$9.51 \cdot 10^{-1}$	$[-2.31, 4.21]$	0.008
		ASP	$-5.69 \cdot 10^{-6}$	$[-2.85 \cdot 10^{-5}, 1.71 \cdot 10^{-5}]$	0.009	$-1.71 \cdot 10^{-6}$	$[-2.50 \cdot 10^{-5}, 2.16 \cdot 10^{-5}]$	0.001
		ACL	$-1.54 \cdot 10^{-5}$	$[-3.77 \cdot 10^{-5}, 6.92 \cdot 10^{-6}]$	0.015	$-9.62 \cdot 10^{-6}$	$[-3.19 \cdot 10^{-5}, 1.27 \cdot 10^{-5}]$	0.003
		AS	$-1.22 \cdot 10^{-3}$	$[-3.53 \cdot 10^{-3}, 1.10 \cdot 10^{-3}]$	0.013	$-1.26 \cdot 10^{-3}$	$[-3.58 \cdot 10^{-3}, 1.06 \cdot 10^{-3}]$	0.016
		TR	$1.79 \cdot 10^{-3}$	$[-1.86 \cdot 10^{-3}, 5.43 \cdot 10^{-3}]$	0.008	$1.75 \cdot 10^{-3}$	$[-1.90 \cdot 10^{-3}, 5.39 \cdot 10^{-3}]$	0.007

Table S6: Linear associations between global metrics and Amyloid Aβ-Cent (Number of subjects = 36) by template and pipeline (slope with 95% CI and R^2). SP: sparsity, MC: mean connectivity, AST: assigned streamlines, GE: global efficiency, ASP: average shortest path, ACL: average clustering, AS: assortativity, TR: transitivity.

**Clinical**	**Pipeline**	**Metric**	**MIITRA**			**MNI**		
			**Slope**	**95% CI**	R^2	**Slope**	**95% CI**	R^2
**Aβ-Cent**	`FAST-ACT`	SP	$1.46 \cdot 10^{-1}$	$[8.94 \cdot 10^{-2}, 2.02 \cdot 10^{-1}]$	0.412	$1.46 \cdot 10^{-1}$	$[8.94 \cdot 10^{-2}, 2.02 \cdot 10^{-1}]$	0.410
		MC	$2.42 \cdot 10^{-1}$	$[1.39 \cdot 10^{-1}, 3.44 \cdot 10^{-1}]$	0.337	$2.45 \cdot 10^{-1}$	$[1.43 \cdot 10^{-1}, 3.48 \cdot 10^{-1}]$	0.339
		AST	$-5.04 \cdot 10^{-2}$	$[-6.99 \cdot 10^{-2}, -3.09 \cdot 10^{-2}]$	0.346	$-5.05 \cdot 10^{-2}$	$[-7.00 \cdot 10^{-2}, -3.10 \cdot 10^{-2}]$	0.350
		GE	$-3.60 \cdot 10^{-1}$	$[-8.18 \cdot 10^{-1}, 9.79 \cdot 10^{-2}]$	0.156	$-3.31 \cdot 10^{-1}$	$[-8.03 \cdot 10^{-1}, 1.42 \cdot 10^{-1}]$	0.133
		ASP	$1.95 \cdot 10^{-6}$	$[-7.32 \cdot 10^{-7}, 4.64 \cdot 10^{-6}]$	0.185	$1.74 \cdot 10^{-6}$	$[-1.18 \cdot 10^{-6}, 4.67 \cdot 10^{-6}]$	0.149
		ACL	$8.80 \cdot 10^{-7}$	$[-1.01 \cdot 10^{-6}, 2.77 \cdot 10^{-6}]$	0.013	$4.01 \cdot 10^{-7}$	$[-1.49 \cdot 10^{-6}, 2.29 \cdot 10^{-6}]$	0.000
		AS	$2.95 \cdot 10^{-4}$	$[1.10 \cdot 10^{-4}, 4.81 \cdot 10^{-4}]$	0.084	$2.74 \cdot 10^{-4}$	$[8.87 \cdot 10^{-5}, 4.60 \cdot 10^{-4}]$	0.077
		TR	$-5.94 \cdot 10^{-4}$	$[-8.63 \cdot 10^{-4}, -3.24 \cdot 10^{-4}]$	0.337	$-5.91 \cdot 10^{-4}$	$[-8.61 \cdot 10^{-4}, -3.22 \cdot 10^{-4}]$	0.336
	`ATR-LOW-ACT`	SP	$1.09 \cdot 10^{-1}$	$[5.21 \cdot 10^{-2}, 1.65 \cdot 10^{-1}]$	0.374	$1.01 \cdot 10^{-1}$	$[4.41 \cdot 10^{-2}, 1.57 \cdot 10^{-1}]$	0.360
		MC	$1.87 \cdot 10^{-1}$	$[8.47 \cdot 10^{-2}, 2.90 \cdot 10^{-1}]$	0.285	$1.50 \cdot 10^{-1}$	$[4.77 \cdot 10^{-2}, 2.53 \cdot 10^{-1}]$	0.227
		AST	$-4.05 \cdot 10^{-2}$	$[-6.00 \cdot 10^{-2}, -2.10 \cdot 10^{-2}]$	0.282	$-4.05 \cdot 10^{-2}$	$[-6.00 \cdot 10^{-2}, -2.10 \cdot 10^{-2}]$	0.285
		GE	$-4.72 \cdot 10^{-1}$	$[-9.88 \cdot 10^{-1}, 4.41 \cdot 10^{-2}]$	0.202	$-4.90 \cdot 10^{-1}$	$[-9.87 \cdot 10^{-1}, 7.48 \cdot 10^{-3}]$	0.189
		ASP	$2.71 \cdot 10^{-6}$	$[-2.55 \cdot 10^{-7}, 5.68 \cdot 10^{-6}]$	0.274	$2.74 \cdot 10^{-6}$	$[-2.77 \cdot 10^{-7}, 5.75 \cdot 10^{-6}]$	0.234
		ACL	$1.26 \cdot 10^{-6}$	$[-6.26 \cdot 10^{-7}, 3.15 \cdot 10^{-6}]$	0.033	$1.90 \cdot 10^{-7}$	$[-1.70 \cdot 10^{-6}, 2.08 \cdot 10^{-6}]$	0.001
		AS	$3.71 \cdot 10^{-4}$	$[1.85 \cdot 10^{-4}, 5.56 \cdot 10^{-4}]$	0.156	$2.44 \cdot 10^{-4}$	$[5.87 \cdot 10^{-5}, 4.30 \cdot 10^{-4}]$	0.084
		TR	$-3.09 \cdot 10^{-4}$	$[-5.79 \cdot 10^{-4}, -3.99 \cdot 10^{-5}]$	0.262	$-2.63 \cdot 10^{-4}$	$[-5.33 \cdot 10^{-4}, 6.13 \cdot 10^{-6}]$	0.231
	`ATR-MED-ACT`	SP	$8.05 \cdot 10^{-2}$	$[2.39 \cdot 10^{-2}, 1.37 \cdot 10^{-1}]$	0.339	$8.06 \cdot 10^{-2}$	$[2.41 \cdot 10^{-2}, 1.37 \cdot 10^{-1}]$	0.334
		MC	$1.29 \cdot 10^{-1}$	$[2.71 \cdot 10^{-2}, 2.32 \cdot 10^{-1}]$	0.239	$1.14 \cdot 10^{-1}$	$[1.18 \cdot 10^{-2}, 2.17 \cdot 10^{-1}]$	0.199
		AST	$-2.06 \cdot 10^{-2}$	$[-4.01 \cdot 10^{-2}, -1.07 \cdot 10^{-3}]$	0.237	$-2.41 \cdot 10^{-2}$	$[-4.36 \cdot 10^{-2}, -4.62 \cdot 10^{-3}]$	0.248
		GE	$-3.23 \cdot 10^{-1}$	$[-6.91 \cdot 10^{-1}, 4.59 \cdot 10^{-2}]$	0.147	$-3.77 \cdot 10^{-1}$	$[-7.63 \cdot 10^{-1}, 1.01 \cdot 10^{-2}]$	0.155
		ASP	$1.60 \cdot 10^{-6}$	$[-2.91 \cdot 10^{-7}, 3.50 \cdot 10^{-6}]$	0.213	$2.00 \cdot 10^{-6}$	$[-3.09 \cdot 10^{-7}, 4.31 \cdot 10^{-6}]$	0.198
		ACL	$7.02 \cdot 10^{-7}$	$[-1.19 \cdot 10^{-6}, 2.59 \cdot 10^{-6}]$	0.006	$-5.15 \cdot 10^{-7}$	$[-2.40 \cdot 10^{-6}, 1.37 \cdot 10^{-6}]$	0.029
		AS	$1.81 \cdot 10^{-4}$	$[-4.45 \cdot 10^{-6}, 3.67 \cdot 10^{-4}]$	0.038	$1.24 \cdot 10^{-4}$	$[-6.14 \cdot 10^{-5}, 3.10 \cdot 10^{-4}]$	0.013
		TR	$-1.92 \cdot 10^{-4}$	$[-4.62 \cdot 10^{-4}, 7.71 \cdot 10^{-5}]$	0.205	$-1.82 \cdot 10^{-4}$	$[-4.52 \cdot 10^{-4}, 8.70 \cdot 10^{-5}]$	0.189
	`ATR-HIG-ACT`	SP	$7.37 \cdot 10^{-2}$	$[1.72 \cdot 10^{-2}, 1.30 \cdot 10^{-1}]$	0.327	$7.07 \cdot 10^{-2}$	$[1.42 \cdot 10^{-2}, 1.27 \cdot 10^{-1}]$	0.300
		MC	$1.22 \cdot 10^{-1}$	$[1.97 \cdot 10^{-2}, 2.25 \cdot 10^{-1}]$	0.236	$9.52 \cdot 10^{-2}$	$[-7.25 \cdot 10^{-3}, 1.98 \cdot 10^{-1}]$	0.171
		AST	$-8.73 \cdot 10^{-3}$	$[-2.82 \cdot 10^{-2}, 1.08 \cdot 10^{-2}]$	0.210	$-1.62 \cdot 10^{-2}$	$[-3.57 \cdot 10^{-2}, 3.31 \cdot 10^{-3}]$	0.263
		GE	$-2.31 \cdot 10^{-1}$	$[-5.43 \cdot 10^{-1}, 8.19 \cdot 10^{-2}]$	0.112	$-3.68 \cdot 10^{-1}$	$[-6.63 \cdot 10^{-1}, -7.27 \cdot 10^{-2}]$	0.185
		ASP	$1.06 \cdot 10^{-6}$	$[-4.91 \cdot 10^{-7}, 2.62 \cdot 10^{-6}]$	0.167	$2.00 \cdot 10^{-6}$	$[2.11 \cdot 10^{-7}, 3.79 \cdot 10^{-6}]$	0.226
		ACL	$6.02 \cdot 10^{-7}$	$[-1.29 \cdot 10^{-6}, 2.49 \cdot 10^{-6}]$	0.003	$-4.93 \cdot 10^{-7}$	$[-2.38 \cdot 10^{-6}, 1.39 \cdot 10^{-6}]$	0.021
		AS	$8.46 \cdot 10^{-5}$	$[-1.01 \cdot 10^{-4}, 2.70 \cdot 10^{-4}]$	0.002	$1.15 \cdot 10^{-4}$	$[-7.08 \cdot 10^{-5}, 3.00 \cdot 10^{-4}]$	0.012
		TR	$-2.20 \cdot 10^{-4}$	$[-4.90 \cdot 10^{-4}, 4.93 \cdot 10^{-5}]$	0.234	$-1.72 \cdot 10^{-4}$	$[-4.41 \cdot 10^{-4}, 9.77 \cdot 10^{-5}]$	0.179
	`WMH-ACT`	SP	$8.17 \cdot 10^{-2}$	$[2.52 \cdot 10^{-2}, 1.38 \cdot 10^{-1}]$	0.319	$8.15 \cdot 10^{-2}$	$[2.50 \cdot 10^{-2}, 1.38 \cdot 10^{-1}]$	0.315
		MC	$1.34 \cdot 10^{-1}$	$[3.12 \cdot 10^{-2}, 2.36 \cdot 10^{-1}]$	0.278	$1.34 \cdot 10^{-1}$	$[3.21 \cdot 10^{-2}, 2.37 \cdot 10^{-1}]$	0.271
		AST	$1.08 \cdot 10^{-3}$	$[-1.84 \cdot 10^{-2}, 2.06 \cdot 10^{-2}]$	0.227	$1.29 \cdot 10^{-3}$	$[-1.82 \cdot 10^{-2}, 2.08 \cdot 10^{-2}]$	0.231
		GE	$1.65 \cdot 10^{-1}$	$[-2.50 \cdot 10^{-1}, 5.80 \cdot 10^{-1}]$	0.021	$1.58 \cdot 10^{-1}$	$[-2.76 \cdot 10^{-1}, 5.92 \cdot 10^{-1}]$	0.017
		ASP	$-9.52 \cdot 10^{-7}$	$[-2.34 \cdot 10^{-6}, 4.40 \cdot 10^{-7}]$	0.039	$-9.57 \cdot 10^{-7}$	$[-2.45 \cdot 10^{-6}, 5.35 \cdot 10^{-7}]$	0.036
		ACL	$1.90 \cdot 10^{-6}$	$[1.49 \cdot 10^{-8}, 3.79 \cdot 10^{-6}]$	0.045	$6.83 \cdot 10^{-7}$	$[-1.21 \cdot 10^{-6}, 2.57 \cdot 10^{-6}]$	0.003
		AS	$3.12 \cdot 10^{-5}$	$[-1.54 \cdot 10^{-4}, 2.17 \cdot 10^{-4}]$	0.005	$3.04 \cdot 10^{-5}$	$[-1.55 \cdot 10^{-4}, 2.16 \cdot 10^{-4}]$	0.006
		TR	$-4.08 \cdot 10^{-4}$	$[-6.77 \cdot 10^{-4}, -1.38 \cdot 10^{-4}]$	0.288	$-4.03 \cdot 10^{-4}$	$[-6.72 \cdot 10^{-4}, -1.33 \cdot 10^{-4}]$	0.281
	`NOACT`	SP	$6.00 \cdot 10^{-2}$	$[3.43 \cdot 10^{-3}, 1.16 \cdot 10^{-1}]$	0.242	$5.87 \cdot 10^{-2}$	$[2.15 \cdot 10^{-3}, 1.15 \cdot 10^{-1}]$	0.233
		MC	$5.37 \cdot 10^{-2}$	$[-4.88 \cdot 10^{-2}, 1.56 \cdot 10^{-1}]$	0.151	$5.51 \cdot 10^{-2}$	$[-4.73 \cdot 10^{-2}, 1.58 \cdot 10^{-1}]$	0.153
		AST	$-6.68 \cdot 10^{-3}$	$[-2.62 \cdot 10^{-2}, 1.28 \cdot 10^{-2}]$	0.115	$-4.73 \cdot 10^{-3}$	$[-2.42 \cdot 10^{-2}, 1.48 \cdot 10^{-2}]$	0.093
		GE	$6.30 \cdot 10^{-2}$	$[-2.19 \cdot 10^{-1}, 3.45 \cdot 10^{-1}]$	0.003	$6.82 \cdot 10^{-2}$	$[-2.05 \cdot 10^{-1}, 3.41 \cdot 10^{-1}]$	0.004
		ASP	$-4.46 \cdot 10^{-7}$	$[-2.53 \cdot 10^{-6}, 1.64 \cdot 10^{-6}]$	0.001	$-5.08 \cdot 10^{-7}$	$[-2.53 \cdot 10^{-6}, 1.52 \cdot 10^{-6}]$	0.002
		ACL	$2.53 \cdot 10^{-7}$	$[-1.63 \cdot 10^{-6}, 2.14 \cdot 10^{-6}]$	0.000	$2.25 \cdot 10^{-7}$	$[-1.66 \cdot 10^{-6}, 2.11 \cdot 10^{-6}]$	0.001
		AS	$4.88 \cdot 10^{-5}$	$[-1.37 \cdot 10^{-4}, 2.34 \cdot 10^{-4}]$	0.005	$5.90 \cdot 10^{-5}$	$[-1.26 \cdot 10^{-4}, 2.45 \cdot 10^{-4}]$	0.000
		TR	$-4.20 \cdot 10^{-4}$	$[-6.89 \cdot 10^{-4}, -1.50 \cdot 10^{-4}]$	0.238	$-4.16 \cdot 10^{-4}$	$[-6.85 \cdot 10^{-4}, -1.46 \cdot 10^{-4}]$	0.231

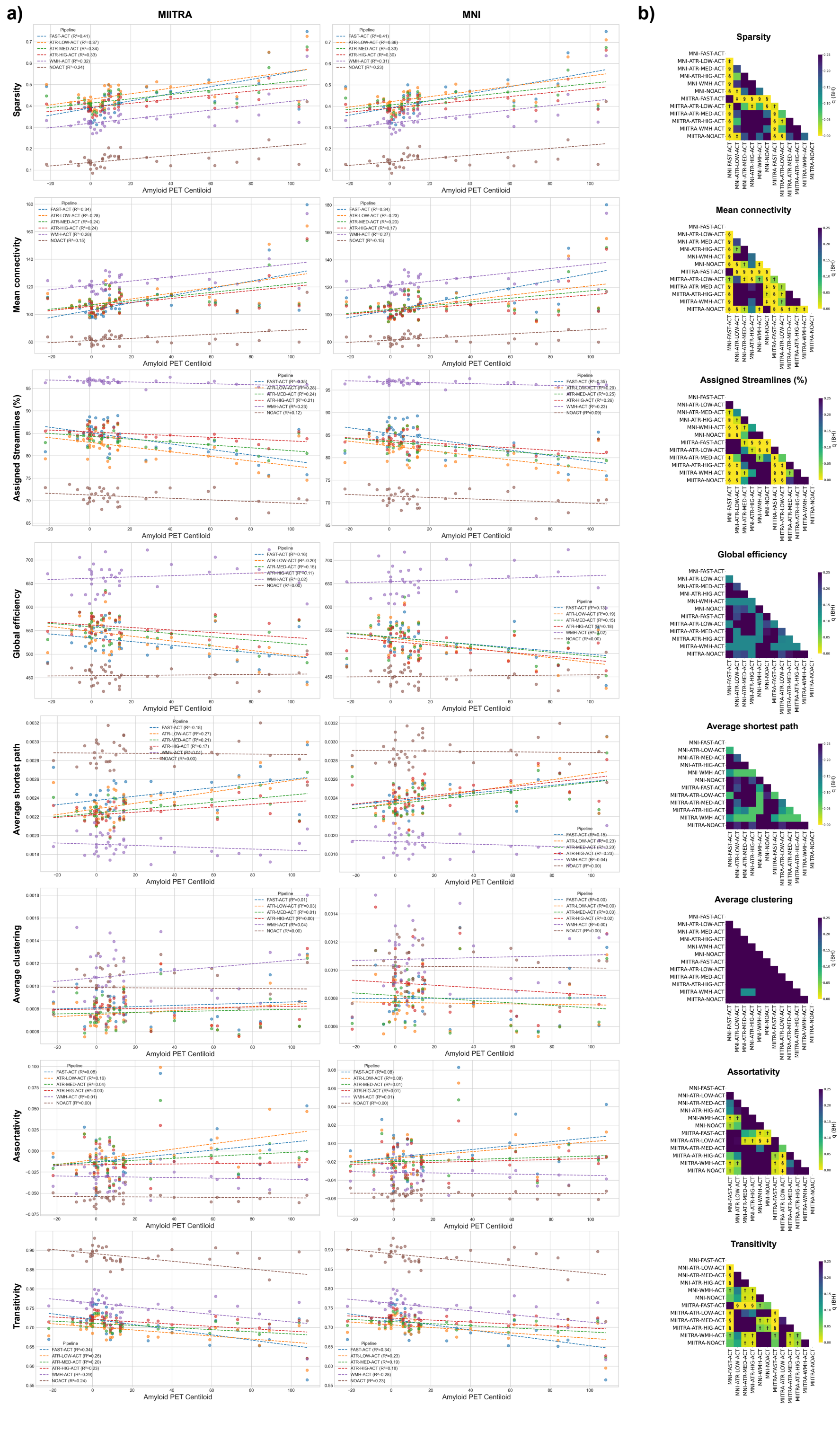

Figure S6: **a)** Scatter plots for seven global metrics (in top to bottom order: sparsity (SP), mean connectivity (MC), assigned streamlines (AST), global efficiency (GE), average shortest path (ASP), average clustering (ACL), assortativity (AS), transitivity (TR) shown separately by template (left: MIITRA; right: MNI) where each point is a subject (x-axis: Aβ-Centiloid; y-axis: metric). Dashed lines indicate per-pipeline linear fits and legends report R^2 for each fit. **b)** Lower-triangular heatmaps of FDR-corrected q-values (Benjamini–Hochberg) for pairwise differences between the slopes estimated in (a). Cells are annotated with significance markers ($^{\dagger}q < 0.05$, $^{\ddagger}q < 0.01$, $^{\S}q < 0.001$).

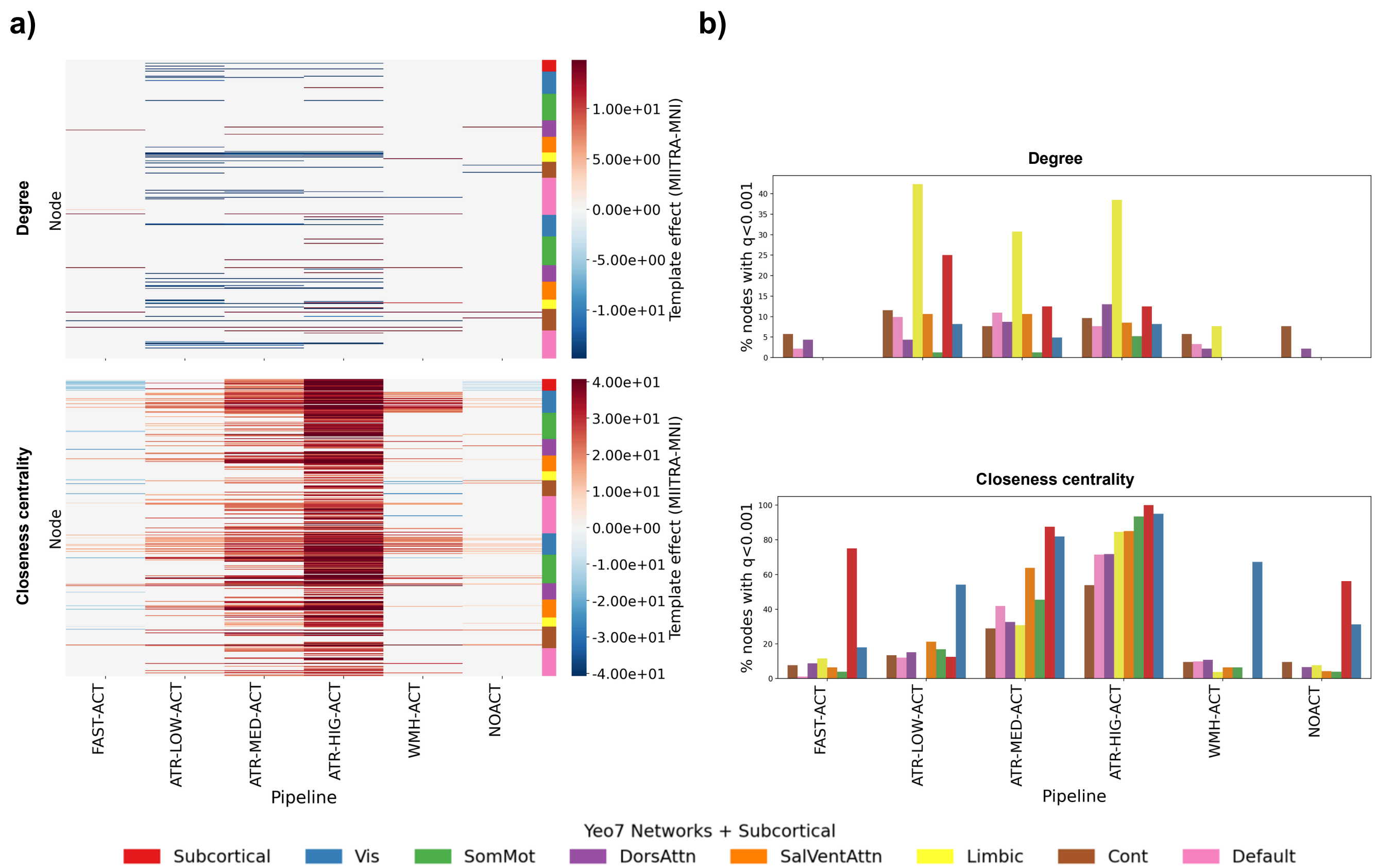

Figure S7: **a)** Heatmaps of the MIITRA–MNI median node effect for four nodal metrics (from top to bottom: degree (DE), closeness centrality (CC)) across pipelines. Nodes are ordered and color-coded by Yeo-7 networks plus the subcortical network (legend at the bottom). Warm/cool colors indicate positive/negative MIITRA–MNI differences. **b)** Bar plots report, for each pipeline and network, the percentage of nodes with a significant MIITRA–MNI effect ($q < 0.001$).
